# Impact of Private Sector Delivery of Quality Care on Maternal, Newborn, and Child Health Outcomes in Low- and Middle-Income Countries: A Systematic Review

**DOI:** 10.5334/aogh.4596

**Published:** 2025-06-20

**Authors:** Samantha R. Lattof, Joe Strong, Blerta Maliqi, Nuhu Yaqub

**Affiliations:** 1 Department of Maternal, Newborn, Child, Adolescent Health and Ageing, World Health Organization, Geneva, Switzerland; 2 Department of International Development, London School of Economics and Political Science, Houghton Street, London, UK

**Keywords:** systematic review, private health sector, maternal health, newborn health, child health, quality of care, low- and middle-income countries, India, Bangladesh, Uganda, Kenya

## Abstract

Evidence regarding the impact of the private health sector on healthcare outcomes is often fragmented. Knowledge gaps remain around the impact of private sector care on health outcomes. This systematic review examines the quality of maternal, newborn, and child health (MNCH) care delivery by private sector providers. The review aims to systematically assess the evidence from studies reporting outcome data on morbidity and mortality among mothers, newborns, and children.

Searches were conducted in eight electronic databases (Cumulative Index to Nursing and Allied Health, EconLit, Excerpta Medica Database, International Bibliography of the Social Science, Popline, PubMed, ScienceDirect, and Web of Science) and two websites and supplemented with hand-searches and expert recommendations. We conducted the searches and application of inclusion/exclusion criteria using the PRISMA method. For inclusion, studies in low- and middle-income countries must have examined at least one of the following primary outcomes: maternal morbidity, maternal mortality, newborn morbidity, newborn mortality, child morbidity, or child mortality. Quantitative and qualitative data were extracted for descriptive statistics and thematic analysis.

Of the 46 studies included, most studies were conducted in India, Bangladesh, Uganda, and Kenya. Thirty-six studies were quantitative, and over one-third implemented a specific intervention that went beyond the broad delivery of quality care in the private sector.

Studies indicated that the outcomes of private sector delivery of MNCH care across health systems were mixed. Studies frequently reported on the utilization of health facilities for the treatment of morbidities. Interventions to improve MNCH care included improved coverage and contracting services, community-based training, and public–private partnerships. Studies often did not provide greater contextual detail, including the complexities and realities of people seeking care across provider types. Future research should disaggregate data on quality of care, as well as describe the methods and specific facility details in their sample.

## Introduction

To achieve the Sustainable Development Goals, including universal health coverage, the public and private health sectors must invest in increasing coverage of interventions and ensuring quality is sustainable at scale. Improving access to effective, high-quality care is critical for saving lives and improving maternal, newborn, and child health (MNCH) [1].

Private healthcare is one of the fastest growing segments of the healthcare system in many low- and middle-income countries (LMICs), and the private sector (i.e. all non-state actors involved in health: profit and not-for-profit, formal and informal, domestic and international) provides an important source of MNCH care. For antenatal care, the private sector accounts for a market share of 13–61% across LMICs, and for delivery care, it accounts for a market share of 9–56% across LMICs [2]. Yet, the evidence about how to sustain and ensure private sector quality care delivery is fragmented, varying significantly across contexts and health systems [3]. This is compounded by the heterogeneity of what constitutes the private sector, which presents significant opportunities as well as complexities in determining how private care providers are engaged [4]. More systematic analyses and approaches are required to understand the impact of private sector care and to effectively engage the private sector in improving MNCH outcomes [5].

A growing body of evidence from systematic reviews and literature reviews has started to examine the intersections between quality of care (QOC), MNCH, and the private sector in LMICs. One systematic review examined strategies to improve the quality of maternal and child health in LMICs [6], but it did not focus on the private sector. Other reviews have focused on particular issues within private sector care (e.g., equity, quality, cost-effectiveness, contracting-out of primary healthcare services) or specific categories of private sector care (e.g., service delivery, financing, child health services) [7–10]. To the best of our knowledge, no systematic review has examined private sector delivery of quality care for MNCH in LMICs.

Since 2019, the World Health Organization (WHO) Department of Maternal, Newborn, Child, Adolescent Health and Aging has been developing an evidence-base that aims to fill gaps around how to effectively engage and sustain private sector involvement in delivering quality MNCH care in LMICs. We examine QOC using the WHO’s six domain framework, in which QOC is effective, safe, people-centered, timely, equitable, and efficient [11, 12]. These components are critical for improved health outcomes and delivering care that meets a person’s needs [13, 14].

As part of this effort and to support a country’s implementation efforts, the Department conducted a systematic review that addresses four primary research questions:

1. How does the provision of quality healthcare by the private sector affect morbidity and mortality among mothers, newborns, and children?
2. How does provision of quality healthcare by the private sector affect the utilization of services by mothers, newborns, and children?
3. How effective and efficient is the private sector at delivering QOC?
4. Among mothers, newborns, and children utilizing health care provided by the private sector, what are their experiences of care? [15]

This study is part of the larger systematic review that examines quantitative, qualitative, and mixed-methods studies addressing the provision of quality MNCH care by private sector providers in LMICs. Our aim in this article is to systematically assess the evidence from studies reporting outcome data on morbidity and mortality among mothers, newborns, and children to answer the first research question:

*How and to what extent does the provision of quality healthcare by the private sector affect morbidity and mortality among mothers, newborns and children?*

It does so by examining outcomes reported in studies of MNCH care that incorporated at least one component of the QOC domains. By better understanding the impact of private sector quality MNCH care, policymakers, healthcare managers and practitioners can better identify and develop best practices for delivering quality MNCH care through engagement with the private sector. Results from complementary analyses on experience of care, QOC, and models and mechanisms for engaging the private sector are presented in separate companion articles.

## Methods

Following guidance in the Preferred Reporting Items for Systematic reviews and Meta-Analyses (PRISMA) Statement for clear and transparent reporting of systematic reviews and meta-analyses [16, 17], we conducted a systematic review that we registered with the PROSPERO international prospective register of systematic reviews (registration number CRD42019143383).

Studies were included if they examined at least one of the following areas: maternal morbidity, maternal mortality, newborn morbidity, child morbidity, and/or child mortality (see Table 1). Further criteria were that the study be published between January 1, 1995 and June 30, 2019, in recognition of the rapid changes in public–private health collaborations during the 1990s [18], be published in English, French, German, or Italian, and be based on or have one study context in LMICs. Studies could be qualitative, quantitative, and/or mixed methods.

**Table 1:** PICOTS criteria used in the systematic review.

PICOTS	
Populations	Pregnant women, mothers, and newborns
Interventions	Delivery of quality maternal and newborn health services by the private sector
Control	Not necessary
Outcomes	Quantitative, qualitative, or mixed-methods data on: maternal morbidity maternal mortality newborn morbidity newborn mortality child morbidity child mortality service utilization experience of care, including respectful care components of quality care (i.e. safety, effectiveness, timeliness, efficiency, equity, people-centered care) secondary outcome: infant and/or child growth
Timeframe	January 1, 1995 to June 30, 2019
Setting	Low- and middle-income countries

To identify relevant studies, we searched eight electronic databases (Cumulative Index to Nursing and Allied Health, EconLit, Excerpta Medica Database, International Bibliography of the Social Sciences, Popline, PubMed, ScienceDirect, and Web of Science) and two websites (Health Care Provider Performance Review and the Maternal healthcare markets Evaluation Team at the London School of Hygiene & Tropical Medicine) (see Table 2 for search terms). In addition to these searches, hand searching based on reference lists, as well as expert-recommended articles, was used to supplement the systematic review. Both peer-reviewed and gray literature were included. The systematic review used a rigorous protocol throughout to guide the process (see [15]). Searches were completed on June 23, 2020.

**Table 2:** Search terms and their combinations.

1. PRIVATE SECTOR	2. QUALITY OF CARE	3. MNCH
private sector	quality	matern*
for-profit		pregnan*
for profit		mother*
public–private		newborn*
private enterprise*		infant*
NGO		child*
non-government*		pediatric*
		paediatric*
		neonat*

Quantitative and qualitative data were extracted from the following categories:

- Background information (e.g., author, date, setting, study objective)
- Intervention background information (e.g., implementing agency, geographic level, study population)
- Intervention details (e.g., intervention recipients, nature of intervention, dimensions of quality care)
- Critical outcomes (both quantitative and qualitative):
  - Maternal morbidity
  - Maternal mortality
  - Newborn morbidity
  - Newborn mortality
  - Child morbidity
  - Child mortality
- Secondary outcome
  - Infant and/or child growth
- Evaluation/study details (e.g., study type, data type, intervention claims, strategy effectiveness, cost data)
- Study quality (qualitative and quantitative)

Studies were extracted by two authors and quality assessed by both authors using quality assessment tools for quantitative and qualitative studies (see [19] for further details). This systematic review focused on the provision of private sector care through the lens of quality care. Quality care was defined as having six domains: efficiency, equity, effectiveness, people-centered care, safety, and timeliness. Studies were only included if they were focused on healthcare that aimed to improve at least one of these components of quality care. Findings specific to each component of QOC will be reported in a separate article.

Due to the high heterogeneity of quantitative outcomes and interventions, meta-analysis was unfeasible for this systematic review. We thus report the study characteristics, outcome measures, and key findings using a narrative approach with tables of descriptive statistics. Outcomes included changes in coverage and the use of different facilities in the treatment of MNCH morbidities, alongside data and evidence relating to changes in the prevalence of morbidities or mortality among key populations. Facility type is included where specified; the heterogeneity of the private sector made facility-based analysis unfeasible. This also means that the systematic review does not seek to make direct comparisons between public and private outcomes unless explicated in included studies. More detailed summary tables, including quality scores, appear in Supplementary Annexes 1 and 2.

## Findings

### Descriptive statistics

**Figure 1:**
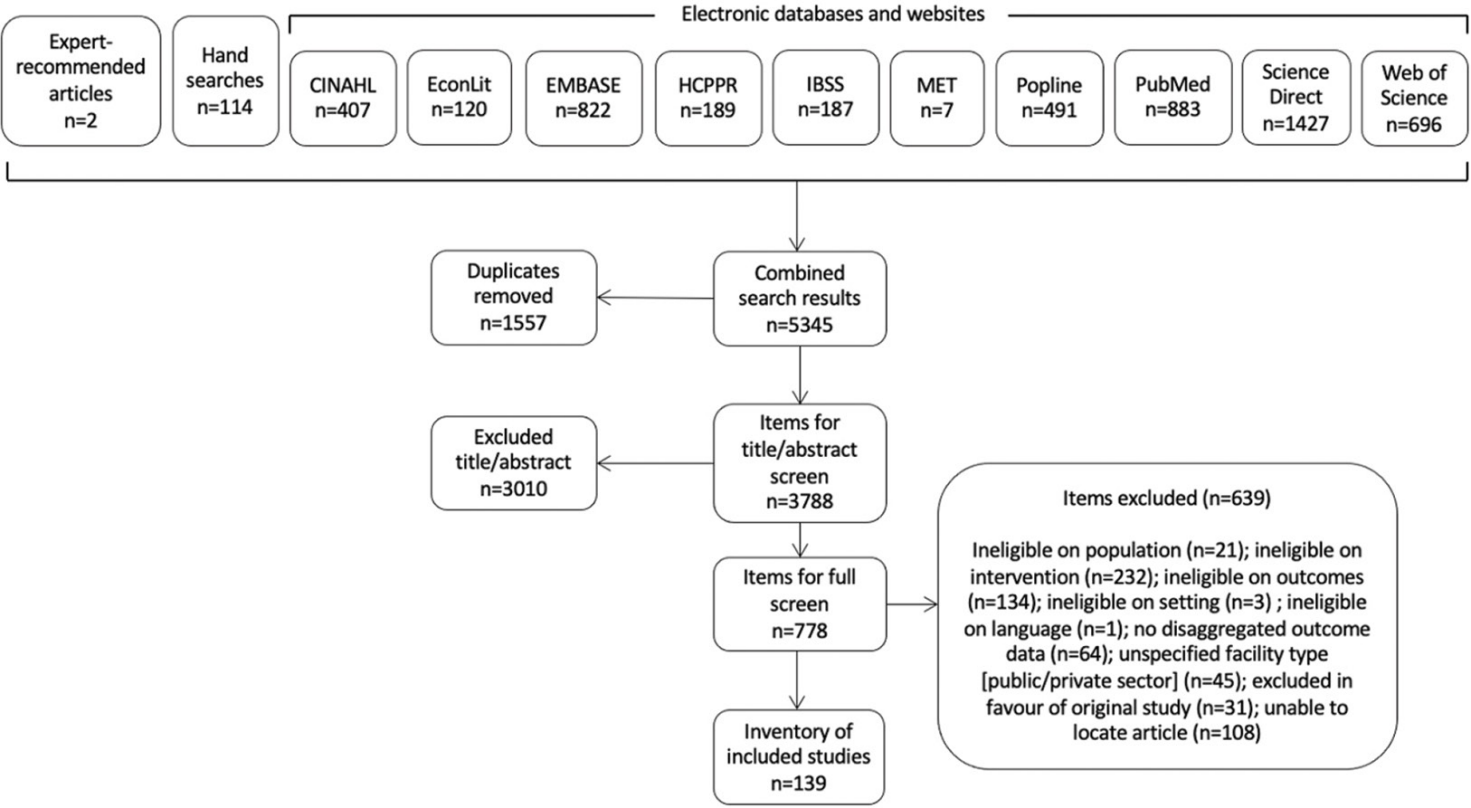
Screening results.

The searches generated 5,345 items for screening (Figure 1). Following the removal of duplicates, 3,788 items remained and were screened for inclusion on the basis of the title and abstract. SRL screened the full texts when exclusion could not be determined on the basis of title and abstract. In total, 139 studies met the inclusion criteria and were included in the broader systematic review.

For the overall systematic review, studies most frequently reported outcome data on QOC (n = 110) followed by experience of care (n = 45) (see [19] for the review of experience of care) (Table 3). Some studies presented multiple relevant outcomes Thus, the total number of data points in Table 3 exceeds the number of studies in the final inventory. The findings in this article focus on the 51 studies that reported outcome data on maternal morbidity, maternal mortality, newborn morbidity, newborn mortality, child morbidity, child mortality, and/or infant/child growth (a secondary outcome).

**Table 3:** Outcomes of included studies.

REPORTED STUDY OUTCOMES	NUMBER OF STUDIES IN THE FINAL INVENTORY THAT REPORT THE OUTCOME
Maternal morbidity	15
Maternal mortality	6
Newborn morbidity	6
Newborn mortality	16
Child morbidity	14
Child mortality	10
Quality of care	110
Experience of care	45
Service utilization	7
Infant/child growth*	9

*Secondary outcome.

Most studies reporting outcome data on maternal, newborn, and child morbidity and mortality were conducted in India (21.6%), Bangladesh (15.7%), Uganda (7.8%), and Kenya (7.8%) (Table 4). Four out of five studies were purely quantitative in nature (Table 5). Nearly two-thirds of studies (56.9%) occurred in countries classified as lower-middle-income. Over one-third of studies (41.2%) implemented a specific intervention that went beyond the broad delivery of quality care in the private sector (58.8%). These interventions were most often single interventions (71.4%) and focused on supply-side factors (57.1%) (Supplementary Annex 1). Interventions targeting learning systems were most common (61.9%) followed by interventions targeting advocacy (47.6%). Additional details of specific intervention studies appear in the thematic analyses below. Summary tables for all MNCH outcomes, including quality assessments, appear in Supplementary Annex 2.

**Table 4:** Included studies by region and country.

REGION/COUNTRY	NUMBER OF STUDIES INCLUDED IN FINAL INVENTORY (%)	NUMBER OF STUDIES EXAMINING MORBIDITY AND/OR MORTALITY AMONG MOTHERS, NEWBORNS, AND CHILDREN (%)	REGION/COUNTRY	NUMBER OF STUDIES INCLUDED IN FINAL INVENTORY (%)	NUMBER OF STUDIES EXAMINING MORBIDITY AND/OR MORTALITY AMONG MOTHERS, NEWBORNS, AND CHILDREN (%)
**Africa**	**49 (35.3%)**	**18 (35.3%)**	**Asia** ^1^	**67 (48.2%)**	**27 (52.9%)**
Angola	1 (0.7%)	—	Afghanistan	2 (1.4%)	—
Côte D’Ivoire	1 (0.7%)	—	Bangladesh	11 (7.9%)	8 (15.7%)
Ghana	1 (0.7%)	1 (2.0%)	China	2 (1.4%)	1 (2.0%)
Ethiopia	2 (1.4%)	2 (3.9%)	Georgia	1 (0.7%)	1 (2.0%)
Kenya	11 (7.9%)	4 (7.8%)	India	30 (21.6%)	11 (21.6%)
Lesotho	1 (0.7%)	1 (2.0%)	Indonesia	2 (1.4%)	1 (2.0%)
Malawi	3 (2.1%)	2 (3.9%)	Iran	2 (1.4%)	2 (3.9%)
Niger	1 (0.7%)	—	Jordan	1 (0.7%)	—
Nigeria	3 (2.2%)	1 (2.0%)	Nepal	4 (2.9%)	2 (3.9%)
Tanzania	3 (2.2%)	1 (2.0%)	Pakistan	6 (4.3%)	1 (2.0%)
The Gambia	1 (0.7%)	—	Philippines	2 (1.4%)	—
Uganda	15 (10.8%)	4 (7.8%)	Sri Lanka	2 (1.4%)	—
Zambia	2 (1.4%)	1 (2.0%)	Turkey	2 (1.4%)	—
Multiple countries	4 (2.9%)	1 (2.0%)			
**Latin America & Caribbean**	**14 (10.1%)**	**4 (7.8%)**	**Oceania** ^1^	**1 (0.7%)**	**—**
Brazil	5 (3.6%)	1 (2.0%)	Papua New Guinea	1 (0.7%)	—
Guatemala	2 (1.4%)	1 (2.0%)			
Haiti	2 (1.4%)	—	**Cross-regional studies**	**8 (5.8%)**	**2 (3.9%)**
Mexico	4 (2.9%)	1 (2.0%)			
Multiple countries	1 (0.7%)	1 (2.0%)	**Total**	**139 (100%)**	**51 (100%)**

^1^ *Asia and Oceania include countries that belong to the following World Bank group classifications: East Asia and the Pacific, Europe and Central Asia, the Middle East, and South Asia.*

**Table 5:** Characteristics of included studies.

CHARACTERISTICS	NUMBER OF STUDIES INCLUDED IN FINAL INVENTORY (%)	NUMBER OF STUDIES EXAMINING MORBIDITY AND/OR MORTALITY AMONG MOTHERS, NEWBORNS, AND CHILDREN (%)
**Methodology**		
Randomized controlled trial	2 (1.4%)	1 (2.0%)
Randomized controlled trial	1 (0.7%)	—
Controlled clinical trial	1 (0.7%)	—
Cohort analytic	10 (7.2%)	4 (7.8%)
Case-control	2 (1.4%)	2 (3.9%)
Cohort (before & after)	7 (5.0%)	2 (3.9%)
Interrupted time series	1 (0.7%)	1 (2.0%)
Qualitative	8 (5.8%)	2 (3.9%)
Mixed methods	21 (15.1%)	9 (17.6%)
Regression	55 (39.6%)	23 (45.1%)
Other	31 (22.3%)	7 (13.7%)
Unclear/not specified	2 (1.4%)	—
**Country income group**		
Low	33 (23.7%)	11 (21.6%)
Lower-middle	75 (54.0%)	29 (56.9%)
Upper-middle	19 (13.7%)	7 (13.7%)
Multiple	12 (8.6%)	4 (7.8%)
**Geographical level**		
National	34 (24.5%)	7 (13.7%)
Sub-national (e.g. state, city)	73 (52.5%)	30 (58.8%)
Local (e.g. village)	7 (5.0%)	1 (2.05)
Health facility	18 (12.9%)	9 (17.6%)
Other	5 (3.6%)	2 (3.9%)
Unclear/not specified	2 (1.4%)	2 (3.8%)
**Study population**		
Pregnant women	11 (7.9%)	4 7.8%)
Women during childbirth	2 (1.4%)	1 (2.0%)
Mothers postpartum	12 (8.6%)	1 (2.0%)
Newborns	13 (9.4%)	3 (5.9%)
Children	9 (6.5%)	5 (9.8%)
Healthcare providers	41 (29.5%)	10 (19.6%)
Parents/child caretakers	4 (2.9%)	3 (5.9%)
Multiple answers from list	26 (18.7%)	11 (21.6%)
Other (e.g., urban poor, married women)	20 (14.4%)	13 (25.5%)
Unclear/unspecified	1 (0.7%)	—
**Publication type**		
Peer-reviewed journal article	103 (74.1%)	33 (64.7%)
Report	27 (19.4%)	13 (25.5%)
Book or book chapter	1 (0.7%)	1 (2.0%)
Other (e.g., conference paper, abstract)	8 (5.8%)	4 (7.8%)
**Implemented a specific intervention beyond the delivery of quality care?**		
Yes	58 (41.7%)	21 (41.2%)
No	81 (58.3%)	30 (58.8%)
**Type of data**		
Quantitative	104 (74.8%)	40 (78.4%)
Qualitative	8 (5.8%)	2 (3.9%)
Both	27 (19.4%)	9 (17.6%)
**Longitudinal data?**		
Yes	45 (32.4%)	19 (37.3%)
No	90 (64.7%)	30 (58.8%)
Unclear/not specified	4 (2.9%)	2 (3.9%)

### Maternal morbidity

Maternal morbidity was among the most reported outcomes (n = 15) with studies reporting morbidity measures during pregnancy, childbirth, abortion, and the postpartum period. While the measures used vary, many studies provided data on where treatment for maternal morbidities was sought. Two themes emerged with regard to obstetric complications other than abortion: (1) maternal complications were commonplace and ranged from 12% to 64% in the public and private sectors; and (2) public facilities generally admitted and treated more women with complications than private facilities. Themes and findings on complications related to abortion are discussed in detail in the following subsection.

Studies examining maternal morbidity often reported on the use of specific services as an outcome of interest. Admissions for dystocia in Indonesian hospitals were similar in public (29.7%) and private (29.9%) facilities; however, public hospitals admitted more women with early pregnancy loss (17.6%) than private hospitals, and private hospitals admitted more women with postpartum hemorrhage (10.1%) than public hospitals [20]. In Bangladesh, two-thirds of the 63.8% of mothers who experienced complications sought treatment from a range of public and private providers, and 31.4% of mothers experienced complications only in childbirth. In another study in Bangladesh, of the 992 mothers who experienced complications at the time of delivery, 5.7% were referred to a public Upazila Health Complex (40.0%), private clinics (33.9%), district hospitals (16.1%), and NGO static clinics (7.2%) [21]. In another study in Bangladesh, a cross-sectional survey of 34 for-profit private hospitals providing maternal and newborn health services reported maternal complications to be 12.5% [22]. This figure is similar to that found in an Indian study on the quality of free delivery care delivered to poor mothers via a public–private partnership: 12% of mothers experienced some complications during pregnancy [23].

While public and private facilities both admitted and treated maternal morbidities, public facilities generally admitted and treated more women with complications than private facilities. In Ethiopia, 152 public health centers treated 5.6 times as many obstetric complication cases resulting from complicated miscarriages (n = 2,512) as 55 private health centers (n = 445) [24]. In Indonesia, public hospitals admitted fewer women with no maternal complications (20.5%) than private hospitals (41.6%) [20]. A study from India found the treatment pattern to vary by location. In urban areas, women experiencing antenatal or postnatal morbidities sought treatment from public and private facilities in equal numbers; however, in rural areas, more women received treatment from public facilities than private facilities [25]. One study compared complications arising from cesarean sections in public and private hospitals. This comparative study from Ethiopia reported a higher maternal morbidity rate following cesarean delivery in government hospitals (7.7%) than in non-government hospitals (0.4%) [26].

### Post-Abortion care

Of the 15 studies that reported outcomes related to maternal morbidities, eight studies reported data on care for complications following an abortion [20, 24, 27–32]. Despite variation in the measures used, the findings show that the private sector can be an important provider of post-abortion care in a safe and timely manner. Both private and public facilities provided abortions and post-abortion care.

Two studies reported outcomes relating to where post-abortion care was sought, and care provided. In Indonesia, public hospitals saw almost twice as many women admitted with life-threatening complications from abortions as private hospitals [20]. Results from a national assessment of safe abortion care services in Ethiopia aggregated maternal deaths with other serious complications of women seeking post-abortion care (e.g., shock, organ, or system failure) [24]. Of women treated for abortion complications at public and private primary-level facilities, 28% of women at public health centers were treated for serious abortion complications, and 20% of women at private health centers were treated for serious abortion complications [24].

Three of the eight studies reporting complications from abortion procedures evaluated interventions that went beyond the generic delivery of quality care by the private sector. In a 2002 report in Kenya, the PRIME post-abortion care program trained private nurse-midwives on 13 key components of post-abortion care, ranging from client-provider interaction and counseling to infection prevention to legal aspects of providing post-abortion care [31]. Based on service data and qualitative findings, a small number of patients required referrals for shock, sepsis, and bleeding; as a result, the authors concluded that private nurse-midwives were able to handle many post-abortion care complications and emergencies [31]. In northern Nigeria, a domestic non-profit organization implemented capacity-building workshops on abortion and post-abortion care [27]. The intervention project’s descriptive analysis showed that only 1.3% of the 2,559 women treated experienced mild to moderate complications, including severe bleeding (n = 24), abdominal pain (n = 8), and anemia (n = 1). Finally, a quasi-experimental study in southwest Bangladesh assessed the feasibility, acceptability, and utility of the safe menstrual regulation and abortion care model among public (n = 44), private-for-profit (n = 22), and NGO not-for-profit (n = 8) facilities [32]. While baseline data revealed that 13% of cases with complications from menstrual regulation and abortion were considered severe, endline data showed that the percentage of cases with severe complications decreased to 11% [32]. At the intervention end, NGOs provided the majority of safe menstrual regulation procedures (93%), compared to public facilities (69%) and private facilities (33%).

Outcomes included the use of referrals in the treatment of abortion complications. Two qualitative studies, both conducted in Tamil Nadu, India, examined the role of government (public) village health nurses in helping their clients obtain abortions in the public and private sectors [29] and women’s experiences of abortion services and abortion safety at government and private facilities [28]. Evidence from the first study highlighted the links between village health nurses and private sector abortion care. In instances of abortion-related complications, village health nurses frequently referred people to private clinics and private nursing homes [29]. This was particularly the case for women who had been rejected from government facilities for care [29].

The second of these studies in India developed a five-point scale for ranking abortion providers/facilities and presented the qualitative findings by three categories of abortion providers: safe (n = 19), intermediate (n = 9), and unqualified and unsafe (n = 8) [28]. While abortion providers/facilities from the public and private sectors were classified as intermediate and unqualified and unsafe, only private providers/facilities were represented in the highly qualified, safe abortion provider/facility group.

### Maternal mortality

Six studies reported outcome data on maternal mortality. Measures of maternal mortality included the number/percentage of maternal deaths, the case-fatality rate for obstetric complications, the odds of a maternal death, mothers who died within 48 hours of transfer, and the estimated contribution to a reduction in the maternal mortality ratio. Of the two studies that reported the number of maternal deaths in public and private hospitals [20, 26], both found proportionally more maternal deaths in public hospitals than in private hospitals. Maternal deaths represented 0.1% of all private hospital admissions (n = 1) and 1.6% of all public hospital admissions (n = 63) in a study from Indonesia [20]. The researchers also noted a significantly higher proportion of obstetric near-misses in public (17.3%) than in private (4.2%) hospitals [20]. During a study in Addis Ababa, Ethiopia, three maternal deaths occurred in the teaching hospitals and no maternal deaths occurred in the hospitals operated by non-governmental organizations (NGOs) [26]. Researchers from a study in Malawi assessed the quality of public and private maternal health systems, finding public facilities to have a higher case-fatality rate for obstetric complications (2.8%) than private facilities (1.5%) [33]. After controlling for complications, public facilities had higher odds of maternal death (odds ratio 1.90, p < 0.000) [33].

Two of the studies reporting maternal mortality evaluated interventions that went beyond the generic delivery of quality care by the private sector. One study evaluated capacity-building workshops on abortion and post-abortion care in northern Nigeria [27]. The domestic non-profit organization implementing the workshops recorded no cases of abortion-related maternal mortality among the 17,009 women treated by the 458 trained providers in 430 private clinics over the intervention’s ten-year implementation [27]. The second intervention introduced emergency response centers in India under a public–private partnership model to provide emergency response services to pregnant mothers, neonates, and other sick people [34]. From 2008 to 2014, data showed that 1.03% of mothers transported died within 48 hours of transport, and 10,542,536 mothers (98.97%) had survived 48 hours after transport. Researchers estimated that the intervention contributed to a 23–35% reduction in India’s maternal mortality ratio [34].

### Newborn and infant morbidity

Six studies reported outcomes related to newborn and infant morbidity. Measures of morbidity among neonates, infants, and newborns included medical conditions (e.g., fever, jaundice) during the first month as stated by the mothers, neonatal complications, complications after delivery, the number of neonates transferred, neonatal admissions, and neonatal patient outcomes. Some studies reported morbidity-related outcomes at birth, while other studies focused on the first week of life, the first month of life, or at 18 months old.

A cross-sectional survey of 34 for-profit private hospitals providing maternal and newborn health services in Bangladesh reported neonatal complications to be 21.5% [22]. In another study in Bangladesh, over half (55.4%) of neonates experienced medical conditions during the first month (e.g., cold/cough, fever, jaundice); nine in ten mothers sought treatment for these illnesses, with private doctors’ clinics delivering 60.1% of facility-based treatments and NGO clinics delivering 14.3% of facility-based treatments [21].

Two studies examined newborn morbidity in studies examining specific interventions beyond the generic delivery of quality care. In India, one study reported on the organization GVK EMRI’s maternal and neonatal transport system, a not-for-profit ambulatory care service that works in collaboration with public health facilities to support access. The study found that the service transferred an increasing number of neonates—from 393 transfers in 2011 to 12,616 transfers in 2014—for conditions including perinatal asphyxia, sepsis, and life-threatening congenital abnormalities [34]. A learning and training intervention for the emergency medical team delivering quality pre-hospital care resulted in the percentage of neonates surviving transport increasing from 85% in 2011 to 94% in 2014 [34]. In Malawi, NGOs built their capacity to scale up HIV-related services, expanded counseling and testing and prevention of mother to child transmission services (PMTCT), improved the quality of services, and increased demand for HIV-related services [35]. While the NGO antenatal clinics recommended that women return for childbirth, the clinics provided women with single-dose Nevirapine after 32 weeks of gestation as prophylaxis for PMTCT in case pregnant women gave birth at home. By 18 months of age, 97.8% of the babies with whom the NGOs followed up (n = 135) tested HIV negative, and three babies tested HIV positive [35].

### Newborn and infant mortality

Sixteen studies reported findings on infant and newborn mortality. This included descriptive studies of care provision in different sectors, clinical observations, and interventions to improve infant and newborn mortality rates. Studies covered various provider types, including public health facilities, NGOs, private health facilities, community health providers, and mission providers.

Some studies reported differences between private and public facility newborn and infant mortality rates as their outcomes of interest, though fewer assessed sector-level differences in capacity to provide complex care. In two linked descriptive studies of neonatal services in Nairobi City Council, Kenya, infant mortality was higher in public sector facilities than in mission and private sector facilities. A cross-sectional review of 31 facilities estimated the infant mortality rate at 16.5% in public facilities compared to 5.9% in mission facilities and 7.3% in private facilities [36]. A review of 1,104 medical records in the same 31 facilities estimated the crude mortality rate of inpatient newborns in public facilities as 8.8%, which was significantly higher than in private sector facilities (3.8%) and mission facilities (2.1%). Importantly, this was only when not adjusted for case-mix or acuity [37]. In clinical observations of 29 private sector and 30 public sector facilities in Uttar Pradesh, India, five neonatal deaths out of 218 deliveries in public facilities and no neonatal deaths out of 64 deliveries in private facilities were recorded [38]. In the study, 51% of private facilities being observed provided recommended neonatal care practices and 39% of public facilities (p = 0.02).

Further studies reported on mortality rates as the outcome of interest when examining private sector delivery of care. Three studies reported on linked interventions that aimed to improve services provided to women of reproductive ages, pregnant women, children, and newborns in Bangladesh through NGO healthcare facilities: The Rural Service Delivery Partnership [39], the Urban NGO Service Delivery Program [40], and the Rural NGO Service Delivery Program [41]. For the Urban NGO Service Delivery Program and the Rural Service Delivery Program, infant mortality was recorded as being lower in non-intervention areas than in intervention areas, while there continued to be an overall trend across regions of decreasing mortality.

Two studies suggested that the increased presence of health systems with higher levels of coordination and collaboration between public and private providers was associated with lower neonatal mortality. An evaluation of a framework for analyzing health infrastructure and infant mortality through a household survey in 1,539 villages in Uttar Pradesh found that the number of private allopathic doctors and in community health centers had a significant, negative effect on infant mortality [42]. A case-control of an intervention in the state of São Paulo, Brazil, that contracted pre-certified non-profit or NGOs to take part in the delivery of health services found that among municipalities with external contracts in the primary health sector there were lower infant and child mortality rates [43].

Some interventions to reduce neonatal mortality engaged the private sector through community education, training programs with local communities as well as with providers themselves. A randomized control trial of the Living Goods and BRAC Community Health Promoters project, in which home visits and household education were conducted, alongside the selling of health products below prevailing retail prices, in 214 villages in Uganda, found infant and neonatal mortality rates decreased compared to control areas [44]. A separate evaluation of the project found that infant mortality fell by 33% and child mortality by 28% in Community Health Promoter villages compared to comparison groups [45].

A number of studies reported on interventions aiming to bridge between the private–public sectors, reporting positive MNCH health-related outcomes. The introduction of skin-to-skin contact training, thermal protection of newborns, neonatal resuscitation, and breastfeeding support, through the USAID-funded, private-sector-led SUSTAIN intervention in 56 MNCH facilities in Georgia, led to reduced infant deaths [46]. The Project Fives Alive! Program in Ghana aimed to improve the coverage, quality, reliability, and patient centeredness of the High Impact Rapid Delivery program through engaging and supporting health workers in public and faith-based (private) facilities. The interrupted time series of mothers, infants, and children under 5 in 25 health centers and two hospitals found the intervention decreased neonatal mortality by a mean of 2.5 to 0.9 per 1,000, and infant mortality from 3.5 to 2.3 per 1,000 [47]. It also highlights the cohesion between public and private care providers.

### Child morbidity

Fourteen studies reported on child morbidity; seven were located in Bangladesh (n = 4) or India (n = 3), five in sub-Saharan Africa (Kenya, Tanzania, Uganda (n = 2), and Zambia), one in Guatemala, and one covering 23 countries globally. Six studies used the prevalence and treatment of diarrhea as a primary area of interest [39–41, 48–50]. The remainder reported on immunization, fevers, respiratory illness, or on a broader range of childhood illnesses.

Child mortality outcomes reported included the increase in coverage; public–private partnerships could be effective in facilitating increased care coverage for children. The 192 public–private urban health centers established by the Commissioner of Family Welfare, India, which aimed to provide basic reproductive and child healthcare, were reported to have facilitated a reduction in childhood illness and an increase in immunization rates to 100% [51]. In Guatemala, a government partnership with 161 NGOs, covering 3,200,000 people, improved the knowledge and use of oral rehydration in the event of children who had diarrhea, though improvements were also reported across districts with different provider types [48]. However, not all public–private partnerships had positive health outcomes. The SkyHealth telemedical program in Bihar, India, which aimed to allow patient consultations and remote assessment through internet connectivity, reported no improvements in child pneumonia or diarrhea [49].

A number of studies reported on the use of private sector care delivery in the treatment of child morbidities, highlighting the mixed role of the private sector in delivering care. Across three linked USAID-funded interventions in Bangladesh, there was minimal impact of the programs on child morbidity [39–41]. In the 2001 Rural Service Delivery Partnership (RSDP) Evaluation Survey, only 0.5% of children with acute respiratory infections sought care from an RDSP provider [39]. For children seeking care for diarrhea, 74.8% sought care from private medical sectors, 16.7% from public facilities, 3.5% at home, with 2.3% using intervention facilities, similar to the 1998 baseline findings [39]. The 2003 Urban NGO Service Delivery Program (NSDP) Evaluation Survey found that private providers in NSDP areas continued to be the most common source of treatment for diarrhea (44% of cases) [40]. This comprised private clinics/doctors (21.3%), pharmacies (16.2%) and then “traditional” doctors (6.7%). The 1% treated by NSDP facilities was the same proportion as in the 2001 baseline, while in the 2005 Rural NGO Service Delivery Program Evaluation Survey, the 2.4% who sought treatment for diarrhea were also similar to prior years, regardless of the intervention [41].

Child morbidity outcomes included care as reported by parents or caregivers. A study of treatment-seeking behaviors among 355 rural and 469 urban households in Choma District, Zambia, found that private facilities were rarely used for the treatment of sick children compared to public facilities, which were used by the majority of respondents in both urban and rural areas. Children who received immunization services from private facilities in a study of healthcare services in Uganda were reported to have fevers twice as commonly as those immunized in public facilities [52]. This difference was not significant in the multivariable analysis conducted. Of the 11% of caregivers who sought care after their child developed a fever, 34% used healthcare workers, with the rest preferring non-facility-based remedies [52].

Finally, outcomes relating to child morbidity included the capacity for different health facilities to treat morbidities. The Health Facilities Survey Report, Bangladesh, reported on the capacity of different health facility types to treat child morbidity. The report indicated that there was a lack of necessary priority medicines (less than 10% across all facilities) for curative child healthcare [53]. More public facilities (93–97%) than NGOs (83%) and private hospitals (68%) provided outpatient curative care for sick children. District and *upazila* public facilities (77%), NGO facilities (57%), and union-level public facilities (55%) are more likely to have integrated management of childhood illness guidelines than private hospitals (26%). Moreover, district and *upazila* public facilities (23%), NGO facilities (17%), and community clinics (10%) are more likely than union-level facilities (5%) and private hospitals (3%) to have all 10 items regarded as necessary to provide child curative care by the WHO. Finally, district and *upazila* public facilities, NGO facilities, and private hospitals (86 to 90%) were more likely to have some hand-cleaning supplies than union-level public facilities (62%) or community clinics (48%).

### Child mortality

Overall, 10 studies reported on child mortality, the majority of which (n = 6) were evaluations of interventions that went beyond the delivery of quality care. Analyses from these studies included randomized controlled trials (n = 2), case control (n = 1), regression analysis (n = 1) mixed-methods analysis (n = 1), and descriptive statistics (n = 1). Interventions engaged with the private sector through community health delivery of non-profits in Uganda [44, 45], to establish and expand quality HIV treatment for children in Botswana, Lesotho, Swaziland, Malawi, Uganda, and Tanzania [54], the contracting of not-for-profit organizations in São Paulo [43], and delivering surgical care in China [55].

The impact of interventions that went beyond the delivery of quality care in the private sector highlighted variations in outcomes. Seventy-five women in Hidalgo, Mexico, whose children died within 90 days with either ARI or diarrhea as a primary or secondary cause, were asked to provide “death narratives” to evaluate the Integrated Management of Childhood Illnesses intervention [56]. Women reported that 27 deaths were linked to the management practices of doctors, including not recognizing the severity of the disease, inadequate treatment, and failure to provide ORS for diarrhea [56]. Private doctors were implicated in 1.8 times the number of deaths compared to public doctors [56]. In both the Rural Service Delivery Program and the Urban NGO Service Delivery Program in Bangladesh, child mortality rates fell by a larger margin in the non-intervention areas than in the intervention areas [39, 40]. Declining mortality in both the intervention and non-intervention areas reflected country-level mortality rate declines over the 15-year period prior to the Rural NGO Service Delivery evaluation [41].

Interventions that incorporated private sector healthcare to reduce child mortality reported outcomes that included increasing community health provider engagement, HIV care access, and access to facility-based care for heart-related illnesses. Children’s Clinical Centers of Excellence, an initiative to establish, expand, and sustain HIV treatment and care for children in Botswana, Lesotho, Swaziland, Uganda, Tanzania, and Malawi, reported reducing the child mortality rate to 3.35 deaths per 100 patient years [54]. Two studies reported on the impact of BRAC Community Health Promoters, who conducted home visits, household education, and sold medicines below retail price in 214 villages in Uganda [44, 45]. Both randomized controlled trials found child mortality reduced by 25% [44] and 27% [45] in the intervention villages compared to comparison villages. The use of external non-profit and non-governmental organizations in primary healthcare delivery in São Paulo state, Brazil, was associated with lower child mortality rates [43].

Among children seeking heart-related care at International Children’s Heart Foundation NGO hospitals, in-hospital mortality was 8.1%, with a reoperation rate of 11.1%, of which 5.7% were returned to the operating room for a bleeding indication. Though the average age of people who received care was 5 years old and the median age 2.8 years old, the age range was reported as 4 days to 60.6 years [57]. Importantly, data were not disaggregated further to establish the extent to which outcomes were related to age, particularly if there were differences between neonates, children, and older individuals.

Another study used 11 observation years of data to report on the outcomes of a partnership between First Hospital of Lanzhou University and Children’s Heart-Link. Children’s Heart-Link provides educational, technical, and medical support to partner health providers. The in-hospital infant mortality rate across this time period was 5.3%. The control equation model indicated that areas of focus of the intervention—including access to care through facility building, equipment and supplies procurement, and availability of trained staff—all led to quality improvements in surgery and were associated with decreased mortality [55].

### Infant and child growth

As a secondary outcome, we extracted relevant data on infant and child growth. Findings from all nine studies reported data relevant to this outcome (Supplementary Annex 3). Six studies reported on infant and child growth, including descriptive statistics and the provision of growth monitoring, with one report on an intervention that specifically trialed growth monitoring technology. Growth monitoring provision varied depending on facility type and sector. In an evaluation of the influence of the Project Fives Alive!, the early implementation phase to improve child survival in Ghana, hospital facilities had a higher percentage of underweight infants than health centers [47].

A number of studies reported on monitoring as the growth-related outcome of interest. The 2014 Health Facility Survey in Bangladesh reported that only 62% of facilities offered growth monitoring, of which 84% offered monitoring across all working weekdays [53]. A total of 55–76% of public and NGO facilities offered services, compared to 20% of private facilities, with urban facilities less likely than rural facilities to monitor child growth [53]. In a comparative study of public health centers and public private cooperative health centers in primary health service delivery programs in Iran, 83.5% of children in public health centers had their growth measured compared to 65.7% in cooperative health centers. However, growth sheets were filled accurately in 69.4% of cooperative health centers compared to 59% in public health centers, and the growth status was reported as favorable in 89.8% of cooperative health centers rather than in 74.3% of public health centers [58]. A program in Guatemala engaged private NGOs to either provide direct care, partner with the Ministry of Health and Social Protection to provide care, or support Ministry of Health providers as direct care providers, to evaluate the most efficient service provision models [48]. Children were more likely to be weighed in direct Ministry of Health (93%) and direct private NGO (100%) provider communities than mixed provider communities (86%) [48].

Highlighting the dynamics of health service use, one study reported on an intervention in Lahore and Rawalpindi, Pakistan, that had an explicit growth monitoring component by providing infantometers and weighing machines [59]. The results indicated a “ripple effect”, in which mothers who were not registered in the intervention trial requested that their children have their growth monitored using the equipment [59].

## Discussion

This systematic review catalogs the provision of quality healthcare by the private sector on morbidity and mortality-related outcomes among mothers, newborns, and children. The review focuses on formal private sector service delivery, with the synthesized evidence also providing insights into health promotion and education. The review highlights the significant variation in the health outcomes of private sector delivery of MNCH care. Making this heterogeneity visible is important for public–private partnerships, highlighting that while overall private sector engagement may complement quality MNCH care, there remain important considerations about where targeted health system strengthening is important. This is particularly the case for maternal mortality and morbidity, where the public sector often remains overburdened by the majority of complex cases.

Private-sector-led interventions that aimed to improve MNCH outcomes could lead to significant health outcome improvements, though not all interventions improved outcomes. Interventions that engaged both the public and private sectors, such as NGOs, to improve coverage and contracting key health services, often had positive improvements on MNCH outcomes across contexts. Training, particularly community-based training and provider training, also improved the recognition and subsequent treatment of care complications, particularly relating to labor. Public–private partnerships can exist to address MNCH outside of formal service delivery, such as training day care workers to promote handwashing and oral hygiene [60]. This review further highlights the importance of examining the contextual factors that may shape whether engaging with the private sector can lead to greater MNCH health coverage that upholds the six core domains of quality care [61].

The provision of necessary medicines, the timeliness of treating complications, and the safety of care, including abortion care, in particular, were all shown to be of considerable importance for morbidity and mortality outcomes in studies included in this review. This highlights the positive health impacts that may be achieved through engaging the private sector in the quality of MNCH care. Included studies indicated that public facilities were frequently burdened with higher numbers of people seeking care, and often were unable to deliver the same MNCH care as private facilities. Often, the delivery of quality of MNCH care across health systems had mixed morbidity and mortality outcomes, regardless of facility type. Studies consistently indicated that the public sector treated significantly more MNCH cases that were often more complex than those in private facilities, which highlights the need to ensure robust support and public facility strengthening across contexts. Private sector MNCH care often complemented coverage but required working in tandem with public sector facilities for referrals and to ensure full health coverage. The public sector was overwhelmingly where COVID-19 cases were treated [62], and thus it may be important to engage the private sector in providing quality MNCH care to ensure health system resilience to potential future health emergencies.

While many studies reported the differences in MNCH outcomes by general facility type—public or private—fewer were situated within the broader context that would help determine whether observed improvements in MNCH are specific to facilities or more generally observed. Private facilities do not operate in isolation and can rather be part of integrated packages incentivized by donors and governments. The nature of these incentives can have important implications for the QOC delivered; evidence in India indicates that the private sector can be demotivated by factors such as delayed reimbursement and administrative burdens, which can impact the success of private–public partnerships [63].

Moreover, people move between different facility types and decisions can vary depending on the health-related care being sought [64]. People’s care-seeking trajectories may mean that they both engage with different sectors within a health system at different stages or manage their healthcare away from the formal health system and occasionally seek additional care. Studies on abortion care-seeking are emblematic of these nuanced trajectories [65]. It is notable, for example, that this review found that the private sector was used where the public sector refused care to women post abortion. The role of stigma is critical to QOC and health outcomes [66], and while private facilities may offer an alternative space for care-seeking, grappling with the larger contextual causes of why people seek care remains important. Many studies in this review did not provide details on the points at which people were seeking care, which may inform the MNCH health outcomes reported. Thus, this systematic review was unable to interrogate the realities and complexities of service use across varied health systems.

There are a number of considerations for this review that are relevant when designing future research and health system evaluations. The searches for this systematic review revealed a number of studies that did not provide disaggregated data for health facilities. Many studies disaggregated data on public and private providers/facilities when presenting background characteristics (e.g., number of days per week the facility is open, percentage of facilities offering postnatal care) but then aggregated public and private sector data when presenting outcomes on MNCH. Some studies included in this systematic review did not present disaggregated data by public or private sector but included one or two general sentences specifying an observed difference between public and private facilities. We included these studies but believe that their contribution could have been stronger if the presented data were disaggregated. Moreover, we acknowledge that advances in MNCH care, particularly in abortion care, have been significant over the period of time this systematic review covers. This systematic review does not assess whether recognized quality care delivered at a specific point in time would still be considered quality care at the time of publishing.

Furthermore, the details of what types of facilities constitute the private sector can often be limited, particularly due to the heterogeneity across the majority of mixed-health systems around the world [4]. Definitions of what constitutes the private sector can differ even within the same Ministry of Health, which further complicates complete provider disaggregation. This systematic review groups different types of facilities and care services under the umbrella of private sector, where there might be differences. Where the private sector is divided between for-profit (formal and informal) and not-for-profit (e.g., NGOs, faith-based organizations), significant variations remain, including whether providers are qualified, the scale of the services, and their integration within the broader health system [67, 68]. This can include differences in governance structures, including the complexities regulating for-profit providers (particularly informal providers) and ensuring alignment among not-for-profit providers [68]. Private providers can be linked to larger corporations or institutions (such as faith-based organizations tied to broader faith-based bureaucracies), while others might operate as small or individual provider entities [4]. Understanding and embracing the complexities of the private sector, as well as further disaggregation of the key actors in this sector in a particular context, will be an important pathway for maximizing the potential of public–private partnerships.

In applying the inclusion/exclusion criteria, we excluded a number of studies presenting data that measured inputs, processes, and outputs. We recognize that measures like the number of health providers trained and the availability of human resources are important components of private sector delivery of quality MNCH care and have a direct impact on our outcome and the impact measures included in this systematic review. We want to move beyond that to look not only at the outcomes of MNCH (experience of care, for example) but the impact of providing MNCH services (reduced morbidity/improved survival).

## Conclusions

This review highlighted many examples of private healthcare contributing to improved health outcomes, but in what form varies substantially by context and thus the literature is not conclusive. Given that MNCH should be of high quality, whether delivered by the public or private sector, policy and decision-makers, and healthcare managers have to explore effective mechanisms that allow for the engagement of both sectors, with objectives that are clearly orientated towards positive and high-quality services and outcomes.

To better understand the impact of private sector quality care on MNCH, the following recommendations are made for future research and policy:

- More evidence is needed on people’s care-seeking trajectories and the way in which they shift between different provider types while accessing health services. This includes accounting for the advances in self-care away from health systems, which may have important implications for how facility-based health outcome data are interpreted.
- Research should locate care-seeking within the social and structural determinants of health, which would allow examination of how decisions over where to seek care, health outcomes, and the ability to deliver QOC intersect with stigma, discrimination, and inequality.
- For researchers who have reported aggregated data on quality care for MNCH, we encourage them to indicate in the manuscript if they have run analyses on the disaggregated data and noticed no significant difference between the two sectors. Otherwise, we encourage these researchers to conduct further analyses in which they disaggregate by public/private sector.
- When describing the study methods, we encourage researchers to provide details on the health facilities/providers in their sample and whether the health facilities/providers belonged to the public sector and/or private sector.
- An updated systematic review should be conducted in the future to account for temporary and permanent changes in quality MNCH provision that occurred with the COVID-19 pandemic.

## Figures and Tables

**Table:** 

LMICs	low- and middle-income countries
MNCH	maternal, newborn, and child health
QOC	quality of care
WHO	World Health Organization

## Data Availability

The data extraction workbook is available on request from SRL (lattofs@who.int).

## Additional Files

The additional files for this article can be found as follows:

Supplementary Annex 1. Summary table of included studies reporting outcome data on MNCH.

Supplementary Annex 2. Intervention characteristics among studies reporting specific interventions that went beyond the generic delivery of quality care.

Supplementary Annex 3. Summary table of included studies reporting outcome data on infant and child growth (n = 9).
