# Supplementary material for: Impact of Private Sector Delivery of Quality Care on Maternal, Newborn, and Child Health Outcomes in Low- and Middle-Income Countries: A Systematic Review
Source: Ann Glob Health. 2025 Jun 20;91(1):35. doi: 10.5334/aogh.4596 (PMC12180434; doi:10.5334/aogh.4596)
Supplement: Supplementary Annex 1. — Summary table of included studies reporting outcome data on MNCH. [file agh-91-1-4596-s1.pdf]

Supplementary Annex 1: Summary table of included studies reporting outcome data on MNCH

Summary table of included studies reporting outcome data on maternal morbidity (n=15)

Author, year [country]	Aim / objective(s)	Setting and population	Intervention Description	Study design	Summary	Quality
**(Abdella, Fetters et al. 2013) [Ethiopia]**	To assess the current availability, distribution, utilisation, and quality of abortion services using the safe abortion care (SAC) model	355 health care providers and 8911 women seeking treatment for abortion complications or induced abortions	Delivery of quality care by the private sector	Mixed-methods	Nationally, a large proportion of women who sought care for obstetric complications, 50% in public primary-level facilities, 75% of women seeking care in private clinics and 43% of women seeking obstetrics or gynaecology services in hospitals, were seeking treatment for complications of an unsafe abortion or a complicated miscarriage. More than one in four women (27%) seeking PAC, regardless of facility type, were already seriously ill based on their symptoms at the time of presentation. Almost three of every four women seeking any type of abortion care (72%) were requesting a safe abortion in the facility. The proportion of women seeking a safe abortion was far higher in primary care facilities, 95% versus only 30% of women seeking abortion care in Ethiopian hospitals.	Moderate
**(Adisasmita, Deviany et al. 2008) [Indonesia]**	To document the frequency, causes, and timing of near miss and deaths in four hospitals in West Java, Indonesia	5669 pregnancy and childbirth related admissions to four hospitals in Pandeglang and serang districts in Banten province, West Java	Delivery of quality care by the private sector	Case study analysis	About a third of admissions were for dystocia, both in public (29.7%) and private (29.9%) hospitals. This was followed by early pregnancy loss in public hospitals (17.6%) and postpartum hemorrhage in private hospitals (10.1%). Abortions represented 14.7% and 7.9% of all admissions in public and private hospitals respectively. Private hospitals admitted more women with normal delivery or non maternal complications such as fetal distress or cord prolapsed than public hospitals (41.6% versus 20.5%)	Weak

Author, year [country]	Aim / objective(s)	Setting and population	Intervention Description	Study design	Summary	Quality
**(Al Haque, Chowdhury et al. 2010) [Bangladesh]**	To test the effectiveness of an integrated evidence-based intervention package addressed skilled birth care, obstetric care, neonatal mortality, and quality of care	Baseline survey of women who have delivered within six months in the Shahjadpur sub-district	Delivery of quality care by the private sector	Baseline data analysis	31.4% of mothers (n=992) had complications at time of delivery, 4.7% were referred for complication management. These referrals were at UHC (40%), private clinics (33.9%), district hospitals (16.1%) and NGO static clinics (7.2%).	Weak
**(Allam, Oruganti et al. 2016) [India]**	A project evaluation of HIV/AIDS services	115 patients accessing HIV testing and counselling and 115 people living with HIV in patient wards	Delivery of quality care by the private sector	Mixed-methods	18 positive pregnant women had institutional deliveries and 93.3% (110) of the women and 95% (112) of children received nevirapine for prevention of parent to child transmission	Weak
**(Aman, Negash and Yusuf 2014) [Ethiopia]**	To compare caesarean delivery practices between government, non-government, and private fee-for-service maternal and child health hospitals	Women who sought caesareans in four study sites in Addis Ababa: Gandhi Memorial Hospital, Tikur Anbasa Specialised Hospital, Saint Paul's Hospital and two private and one non-governmental, maternal and child health fee-for-service hospitals.	Delivery of quality care by the private sector	Case study analysis	The reported maternal morbidity rate was higher in government hospitals compared to non-governmental hospitals, 37 (7.7%) and 2 (0.4%), P<0.05, the common morbidities identified being, wound infection 17 (45.9%), puerperal sepsis 15 (40.5%), and post-partum haemorrhage 3 (8.1%)	Weak
**(Anwar, Begum et al. 2016) [Bangladesh]**	To explore the structural and outcome dimension of quality of services as part of	1343 case records were reviewed across 34 surveyed facilities	Delivery of quality care by the private sector	Cross-sectional survey analyses	High caesarean delivery rate (80%) without supporting partograph (<1%) denote poor performance on evidence-based practice. Irrespective of mode of deliveries, majority of them (67%) took place after 2pm. Reported maternal was 12.5%	Weak

Author, year [country]	Aim / objective(s)	Setting and population	Intervention Description	Study design	Summary	Quality
	implementation research					
**(Audinarayana 2008) [India]**	To review the use of private and public healthcare services among married women in Tamil Nadu through a review of data	Currently married women aged 15-44 in Tamil Nadu	Delivery of quality care by the private sector	Rapid data and literature review	From the literature review: one study found that among 314 women in urban slums and 273 women from three rural settlements (all Scheduled Castes), in Coimbatore district revealed that women who suffered with antenatal morbidity (for the last three episodes of illness) sought treatment equally from government and private facilities irrespective of their place of residence.	Moderate
**(Huda, Ahmed et al. 2015) [Bangladesh]**	To adapt and implement a set of process indicators, bsaed on the safe abortion model (SAC), to supplement the tools and indicators for monitoring emergency obstetric care interventions	74 registered health facilities that provide menstrual regulation, legal abortion, and / or post-abortion care services in Jessore	Training of staffs from participating facilities on the new SMRAC model and introduction of the tool in all selected facilities. Regular visit of project staff to the facilities for monitoring and helping the service providers to use the SMRAC model (May-July 2009).	Quasi-experimental	During the baseline 13 % of all cases with MR/abortion complications were considered severe, while during endline, the percentage went down to 11 %	Weak
**(Karki, Ojha and Rayamajhi 2009) [Nepal]**	To conduct a baseline survey on functioning of existing abortion services in three pilot districts of Nepal	16 comprehensive abortion care centres in three districts	Delivery of quality care by the private sector	Healthcare centre observations	1.98% of cases at MSI were recorded as having minor complications - of which all had come with either incomplete abortion or slight vaginal bleeding. No other sites recorded complications	Weak
**(Nelson, Corbett et al. 2002) [Kenya]**	To assess the PRIME post-abortion care	155 nurse midwives at 120 facilities across six districts of the three pilot project provinces	An intervention aimed at training private nurse-midwives, focused on 13 key components: introduction	Mixed-methods	Nurse midwives were able to handle a variety of complications and emergencies, evidenced by the small number of referrals. Referrals that	Weak

Author, year [country]	Aim / objective(s)	Setting and population	Intervention Description	Study design	Summary	Quality
	programme in Kenya		and clarification of values, client-provider interaction and counselling, management of complications from unsafe or incomplete abortion, MVA procedures, infection prevention, pain management, postabortion family planning counselling and method provision, STI/HIV management, record keeping, legal aspects of providing PAC services, peer supervision, community outreach, performing practical procedures.		were made were mostly for shock, sepsis, and profuse bleeding	
**(Ramachandar and Pelto 2002) [India]**	To report on abortion services and the role of government village health nurses in assissting women to obtain abortions	42 village health nurses from 10 sterilisation/MTP camps in different primary health centre locations	Delivery of quality care by the private sector	Qualitative interviews	Post-abortion complications of varying degrees of severity are still common and are found in both the government and private services. Many of these cases are managed by VHNs, often through referral to private clinics. Timely interventions appear to have reduced the risks from abortion complications.<br><br>Several of the VHNs narrated recent cases of post-abortion complications arising in government sterilisation/MTP camps and hospitals. In one camp we observed, five or six cases were reported to us by VHNs that involved severe bleeding due to incomplete D&C.<br><br>Post-abortion complications were reported to have reduced considerably.	Weak

Author, year [country]	Aim / objective(s)	Setting and population	Intervention Description	Study design	Summary	Quality
**(Ramachandar and Pelto 2004) [India]**	To report on abortion services and the role of government village health nurses in assissting women to obtain abortions	97 married women who had had abortions within the previous six months and 18 village health nurses	Delivery of quality care by the private sector	Qualitative interviews	Two-thirds of the women reported few or no problems following abortion. Morbidity is based on self-report and was considered to remain unacceptably high, with 13 women reported severe complications, 16 moderate, and 68 few or none.	Weak
**(Vora, Saiyed and Mavalankar 2018) [India]**	To determine the quality of free delivery care, examine the differences in the quality of care between public sector facilities and accredited private sector facilities	1616 pregnant women from selected districts	Delivery of quality care by the private sector	Descriptive statistics and chi-squared tests	Only 12% of mothers had any complications during their pregnancy.	Weak
**(Rahman, Rob and Kibria 2009) [Bangladesh]**	To develop a system to distrbute vouches for maternal healthcare services, identify private and non-government organisation facilities that can provide quality maternal health services to poor pregnant	436 pregnant women / mothers for a survey in the two unions of Nabiganj Upazila of Habiganj district; 15 in-depth interviews with women	The intervention provided a 3.5-day orientation for service providers, capacity building of service providers and fieldworkers including three-week training in partnership with Population Council, health facility strengthening to enable the provision of quality services, flipcharts and educational materials to create awareness of the intervention activities amongst communities.	Cohort pre and post	There were significant reductions in complications across all three phases – during pregnancy, no complications increased from 26.8% to 75.4%; delivery, no complications increased from 40.8% to 75.4%, and postpartum, no complications increased from 43.8% to 69.8%.	Weak

Author, year [country]	Aim / objective(s)	Setting and population	Intervention Description	Study design	Summary	Quality
	women, increase the capacity of service providers in offering antenatal care, delivery and postnatal care, improve the level of utilization of antenatal care, delivery, and postnatal care					
**(Ogu, Okonofua et al. 2012) [Nigeria]**	To report outcomes of the training offered to private medical doctors in northern Nigeria, and the effects of the trianing on provision of abortion and postabortion care	1297 participants, including 458 doctors and 839 nurses and midwives, across 8 states in Nigeria	The project consisted of a needs assessment study, development and pretesting of capacity-building modules, capacity building workshops, service delivery, and monitoring and evaluation of patients treated by trained providers	Descriptive statistics and survey data	Of the 2559 treated women, only 33 (1.3%) experienced mild to moderate complications: severe bleeding in 24 cases, abdominal pain in 8 cases, and anemia in 1 case. All cases of severe bleeding responded promptly to oral and/or vaginal administration of misoprostol and to the administration of intravenous fluids.	Weak

Summary table of included studies reporting outcome data on maternal mortality (n=6)

Author, year [country]	Aim / objective(s)	Setting and population	Intervention Description	Study design	Summary	Quality
**(Abdella, Fetters et al. 2013) [Ethiopia]**	To assess the current availability, distribution, utilisation, and quality of abortion services using the safe abortion care (SAC) model	356 health care providers and 8911 women seeking treatment for abortion complications or induced abortions	Delivery of quality care by the private sector	Mixed-methods	Data for mortality and morbidity complications not disaggregated, but complications that included death were reported at hospitals (n=1469), public health centres (n=1266), private health centres (n=3060)	Moderate
**(Adisasmita, Deviany et al. 2008) [Indonesia]**	To document the frequency, causes, and timing of near miss and deaths in four hospitals in West Java, Indonesia	5670 pregnancy and childbirth related admissions to four hospitals in Pandeglang and Serang districts in Banten province, West Java	Delivery of quality care by the private sector	Case study analysis	One maternal death was reported in the private hospitals (0.1% of all admissions), compared to 63 (1.6% of all admissions) in the public hospitals. Maternal mortality, was highest among non-obstetric admissions (13.5%) followed by hypertensive diseases (4.7%), ante partum (2.5%) and postpartum haemorrhage (2.0%)	Weak
**(Aman, Negash and Yusuf 2014) [Ethiopia]**	To compare caesarean delivery practices between government, non-government, and private fee-for-service maternal and child health hospitals	Women who sought caesareans in four study sites in Addis Ababa: Gandhi Memorial Hospital, Tikur Anbasa Specialised Hospital, Saint Paul's Hospital and two private and one non-governmental, maternal and child health fee-for-service hospitals.	Delivery of quality care by the private sector	Case study analysis	There were three maternal deaths reported in the teaching hospitals but none in the non- governmental hospitals during the study period	Weak
**(Kumutha, Rao et al. 2015) [India]**	To describe the GVK EMRI intervention	41,476 personnel across 15 states and 2 union territories	An emergency response centre was established in the capital city of each state and union territory to provide a toll-free number for people seeking emergency care, which assigns ambulance services and emergency medical	Literature review and descriptive statistics	If the transported mother was alive at 48 h of transfer, she was considered as a life saved. Analysis of the 48 h follow-up data from2008 to 2014 showed that 98.97% of mothers were alive at 48 hours after transfer (10,542,536 mothers) and the rest had died within 48 h. The percentage of maternal lives saved in 2014 (99.39%) showed a trend towards improvement when compared	Weak

			teams for quality pre-hospital care.		with the percentage in 2008 (98.28%)<br><br>The effective reach and specific focus on pregnancy-related emergencies by the institute are estimated to contribute to a 23-35% reduction in the MMR in India, as analyzed by Jena et al.	
**(Sharan, Ahmed et al. 2012) [Malawi]**	To examine the quality of maternal health systems and the probability of survival at public and private health facilities in Malawi	21,476 patient records from 86 facilities (36 hospitals and 50 health centres) from 10 districts	Delivery of quality care by the private sector	Descriptive statistics and regression analyses	The case-fatality rate for obstetric complications was higher at public facilities (2.8%) as compared to private facilities (1.5%). The odds of a maternal death was also higher at public facilities (OR 1.90, p < 0.000) after controlling for the case mix of complications	Weak
**(Ogu, Okonofua et al. 2012) [Nigeria]**	To report outcomes of the training offered to private medical doctors in northern Nigeria, and the effects of the training on provision of abortion and postabortion care	1298 participants, including 458 doctors and 839 nurses and midwives, across 8 states in Nigeria	The project consisted of a needs assessment study, development and pretesting of capacity-building modules, capacity building workshops, service delivery, and monitoring and evaluation of patients treated by trained providers	Descriptive statistics and survey data	A total of 17,009 women were treated by 458 trained providers in 430 private clinics over the 10 years of the project (approximately 2100 women annually). No case of abortion-related maternal mortality was recorded	Weak

Summary table of included studies reporting outcome data on newborn morbidity (n=6)

Author, year [country]	Aim / objective(s)	Setting and population	Intervention Description	Study design	Summary	Quality
**(Al Haque, Chowdhury et al. 2010) [Bangladesh]**	To test the effectiveness of an integrated evidence-based intervention package addressed skilled birth care, obstetric care, neonatal mortality, and quality of care	Baseline survey of women who have delivered within six months in the Shahjadpur sub-district	Delivery of quality care by the private sector	Baseline data analysis	More than half (55.4%) of the neonate experienced some kind of medical condition during the first month as stated by the mothers. The common illness in the first month was cold/cough, fever, difficulty in breathing; jaundice to name a few and almost 92.2% of the mothers sought treatment for the illness. Nearly half 49.2 % of them had treatment for illness from	Weak

Author, year [country]	Aim / objective(s)	Setting and population	Intervention Description	Study design	Summary	Quality
					Homoeopathic doctors/village doctors and MBBS doctors accounting for 26%. Facility wise treatment was taken in private/doctors clinic 60.1%, home 22.3% and 14.3% in NGO static clinics.	
**(Anwar, Begum et al. 2016) [Bangladesh]**	To explore the structural and outcome dimension of quality of services as part of implementation research	1343 case records were reviewed across 34 surveyed facilities	Delivery of quality care by the private sector	Cross-sectional survey analyses	Neonatal complications were 21.5%	Weak
**(Kumutha, Rao et al. 2015) [India]**	To describe the GVK EMRI intervention	41,476 personnel across 15 states and 2 union territories	An emergency response centre was established in the capital city of each state and union territory to provide a toll-free number for people seeking emergency care, which assigns ambulance services and emergency medical teams for quality pre-hospital care.	Literature review and descriptive statistics	The number of neonates transferred during the period of four years from 2011 to 2014 had increased by 32 times (393 transfers in 2011 to 12,616 transfers in 2014). The majority of the neonates transferred were male (60.8%) and a higher proportion (67.9%) of term neonates were transported. The reasons for transfer included perinatal asphyxia, sepsis and major life-threatening congenital anomalies. Of the neonates transferred, 43.5% were in the low-birth-weight category; 64% of the neonates were critically ill. The majority of the babies (80%) reached the facility in a stable clinical condition: normal temperature, stable blood sugar, airway, breathing stabilized, and circulation maintained. Of the neonates transported, 85% survived in 2011 and this increased steadily to 94% in 2014 (difference significant)	Weak
**(Murphy, Gathara et al. 2018) [Kenya]**	To describe the provision of and access to inpatient neonatal services within Nairobi City County, and to explore access and evaluate readiness of public, private not-for-profit, and private-for-profit sector facilities	31 facilities in Nairobi City County that provie inpatient neonatal care	Delivery of quality care by the private sector	Cross-sectional	21/31 of facilities providing INC were not able to offer an adequate level of care for common severe newborn illnesses due to inadequacies in structural capacity, or staffing or because they likely had too few admissions per year (<50) to sustain knowledge and skills. Nine (9/21; 78 admissions) of these facilities met the criteria of inadequacy for all three of these features. The 10/31 facilities (10,176 admissions) meeting minimal service criteria included four public, three mission, and three private sector facilities, though	Weak

Author, year [country]	Aim / objective(s)	Setting and population	Intervention Description	Study design	Summary	Quality
					in the busiest of these 10 facilities considerable challenges to the provision of quality care still exist, particularly low nurse to patient ratios and overcrowding	
**(Murphy, Gathara et al. 2018) [Kenya]**	To report on the quality of the process of care delivered to small and sick inpatient newborns across diverse facility settings in Nairobi	33 facilities in Nairobi City County that provie inpatient neonatal care	Delivery of quality care by the private sector	Retrospective review of medical records	Admission diagnosis was documented for 87.0% (1029/1183) of newborns. The most common admission diagnoses were respiratory distress (35.2%), birth asphyxia (32.4%), preterm birth (24.6%), severe infection (18.9%) and jaundice (12.9%). Congenital malformations (3.1%), large for gestational age (3.0%) and dehydration (2.6%) were less common.	Weak
**(Save the Children 2007) [Malawi]**	To provide technical assistance and sub-grants to 15 Malawian non-government organisations (NGOs) to build their capacity to scale up HIV related services.	15 NGOs	Fifteen local partner NGOs were supported between 2003 and 2007. Support included technical assistance in the development of proposals and budgets, capacity building, management information system improvement, resource mobilisation, community mobilisation training, and behaviour change programmes.	Mixed-methods	A total of 1,157 pregnant women, and 603 of their babies were provided with a complete course of ARV prophylaxis in the project period.<br><br>Utilization of labor and delivery services also increased due to the provision in the national PMTCT scale up plan in which the government has agreed a memorandum of understanding with NGOs to pay for maternity services. Generally, this was single dose Nevirapine. At most sites, women were provided with the Nevirapine at their antenatal clinic after 32 weeks gestation in case they delivered at home, though all pregnant women were encouraged to return for a service provider-assisted delivery. Of the 135 babies followed up at 18 months by four of the NGOs (excluding data from Nkhoma), 132 (97%) babies tested HIV negative and three tested HIV positive. All were exclusively breastfed, with early cessation at six months.	Weak

Summary table of included studies reporting outcome data on newborn mortality (n=16)

Author, year [country]	Aim / objective(s)	Setting and population	Intervention Description	Study design	Summary	Quality
**(Angeles, Hutchinson and Khan 2003) [Bangladesh]**	To collect information on and monitor changes in USAID performance indicators, and to conduct an evaluation of the impact of the RSDP programme on the health of the catchment area population	Women ever married, aged 10-49 years: 9769 in the intervention areas, and 3176 in the comparison areas	Delivery of quality care by the private sector	Cohort analytic	The infant mortality rate in RSDP areas for the 5-year period preceding the survey was 77 deaths per 1,000 live births, while the child mortality rate was 28.6 deaths per 1,000. These rates are higher than the rates in non-RSDP areas – 70.5 and 24.1 deaths per 1,000 live births respectively. The 10-year period infant mortality rates in RSDP areas were highest in Sylhet (105.3) and Dhaka (98.7) divisions and lowest in Chittagong division (67.3).	Moderate
**(Angeles, Lance et al. 2005) [Bangladesh]**	To collect information about knowledge, awareness, and use of services related to family planning, maternal and child health, provided by the project intervention	Women ever married, aged 10-49 years: 5691 in the intervention areas, and 4201 in the comparison areas	Delivery of quality care by the private sector	Cohort analytic	The infant mortality rate in NSDP common clusters was nearly the same in 2003 (55 deaths per 1,000 live births) as in 2001 (54 deaths), a result mirrored in the full sample. In non-NSDP areas, mortality (particularly infant) fell by a larger margin.	Weak
**(Angeles, Lance and Khan 2006) [Bangladesh]**	To measure changes in the USAID performance indicators since the mid-project evaluation of the RSDP programme	7652 women from the intervention project areas and 4418 from the non-intervention project areas	Delivery of quality care by the private sector	Cohort analytic	The infant mortality rate in NSDP areas for the five-year period preceding the survey was 57 deaths per 1,000 live births, down from 73 deaths in 2003, and 77 in 2001. The infant mortality rate (62.2 deaths per 1,000 live births) was lower in non-NSDP areas. For the 10-year period preceding the survey, the infant mortality rate for the poorest in project areas was 76.3 (against 63.7 for the full sample in project areas). The 10-year period infant mortality rates in NSDP areas were highest in Dhaka (77.3) and lowest in Chittagong (67.3) division. In both NSDP and	Moderate

Author, year [country]	Aim / objective(s)	Setting and population	Intervention Description	Study design	Summary	Quality
					non-NSDP areas, mortality rates have declined significantly over the past 15 years.	
**(Bhargava, Chowdhury and Singh 2005) [India]**	To create and evaluate a framework for analysing health infrastructure, contraceptive use, and infant mortality	Household survey in 1539 villages in Uttar Pradesh	Delivery of quality care by the private sector	Regression analyses	The average number of private allopathic doctors and the average number of staff trained in family planning methods in community health centres were estimated with negative signs that were significantly associated with chances of infant mortality at the 5 and 10% levels, respectively. Approximately 25% of the women received ante-natal care and 85% of the babies were delivered without qualified personnel.	Moderate
**(Bjorkman-Nykvist, Guariso et al. 2015) [Uganda]**	To asses the impact of non-profit entrepreneurial models of community health delivery	8119 households in 214 villages across 10 districts	A cluster-randomized controlled trial from 214 rural villages in 10 districts in Uganda. In treatment villages, Living Goods and BRAC Community Health Promoters conducting home visits, educating households on essential health behaviours and selling preventive and curative health products at 20-30% below prevailing retail prices were deployed over a three-year period (2011- 2013)	Randomized controlled trial	The intervention reduced under-five mortality rate by 25%. The effects are of the same order of magnitude for infant mortality (< 1 year) and neonatal mortality (< 1 month), although the effect is less precisely estimated for neonatal mortality.	Weak
**(Greve and Schattan Ruas Pereira Coelho 2017) [Brazil]**	To explore the impact of contracting out not-for-profit organisations in the state of São Paulo	Employees on external contracts working in primary health care in 206 municipalities in the state of São Paulo	Intervention contracted pre-certified non-profit or non-governmental organizations to take part in the delivery of health care services	Case-Control	Municipalities implementing external contracts in the primary health care sector had lower infant and child mortality rates and lower hospitalization rates for preventable and respiratory diseases	Weak
**(J-PAL Policy Briefcase 2019) [Uganda]**	To evaluate the Community Health Promoter program by BRAC Uganda	Community health promotors in 214 villages	Comprehensive training on health education and business skills for community health promotors, in order to provide	Randomized controlled trial	Infant mortality fell by 33 percent and neonatal mortality decreased by 28 percent in CHP villages, relative to the comparison group	Weak

Author, year [country]	Aim / objective(s)	Setting and population	Intervention Description	Study design	Summary	Quality
			home visits, health education, basic medical advice and treatment, and referrals to nearby clinics, as well as selling health-related commodities.			
**(Jenkins, Castaneda et al. 2014) [various (17 countries)]**	To evaluate the International Quality Improvement Collaborative for Congenital Heart Surgery in Developing World Countries	Healthcare institutions that self-identified as performing congenital heart surgery (n=28)	Webinars targeted 3 key drivers: safe perioperative practice, infection reduction, and team-based practice.	Risk-adjusted outcomes	The unadjusted in-hospital mortality rate was 6.3%. The 30-day mortality rate was 7.4% among sites reporting 30-day mortality data for at least 90% of surgical cases (7 sites in 2010, 12 sites in 2011, 19 sites in 2012)	Moderate
**(Maru, Maru et al. 2017) [Nepal]**	To describe the impact of care reforms on women and children's health	Government payments to pregnancy and delivery care providers in Achham district	Possible and the Ministry of Health pioneered the public private partnership to deliver high quality, affordable care. The partnership's CHWs provide three core functions: surveillance of conditions in the community, triage, referral and care coordination with facilities, and community-based counselling, education, and diagnosis.	Pre-post census analysis	Infant mortality decreased from 18.3 to 12.5, but this was not significant.	Moderate
**(McIntosh, Grabowski et al. 2015) [Lesotho]**	To compare measures of capacity, utilization, clinical quality, and patient outcomes before and after the implementation of a health care public-private partnership	36 key informant interviews with healthcare providers and ministry workers; observational data from healthcare facilities.	Delivery of quality care by the private sector	Mixed-methods	In the public-private partnership-managed hospital, 46% of neonates in the neonatal intensive care unit (NICU) weighed less than 1,500 grams (the usual definition of very low birthweight), and 69.8% of these patients survived to discharge. The government-managed hospital did not have a NICU, so this measure was not assessed at baseline. Given the medical complexity of providing care to these infants, however, it is likely that without a NICU, most infants would not have	Weak

Author, year [country]	Aim / objective(s)	Setting and population	Intervention Description	Study design	Summary	Quality
					survived to discharge at the government-managed hospital	
**(Mendez and Associates 2014) [Georgia]**	To evaluate the flexibility, effectiveness, impact, contribution, satisfaction and sustainability of an MNCH facility intervention	56 health facilities providing maternal, newborn and child health throughout Georgia	The SUSTAIN intervention was intended to extend and sustain MNCH services through capacity building of networks of private facilities and providers, as well as adding new technical approaches including management of third stage labour, preeclampsia and eclampsia, postpartum haemorrhage, pregnancy-induced hypertension, and neonatal resuscitation.	Quasi-experimental	Neonatologists specifically mentioned that introduction of skin to skin contact training and thermal protection of newborn, neonatal resuscitation and breastfeeding support resulted in many positive neonatal outcomes and reduced infant deaths. Examples of positive clinical outcomes that they mentioned included reduced complications of PPH, more babies adequately and effectively resuscitated, and ability to intervene by tracking progress of labor using the partograph.	Weak
**(Murphy, Gathara et al. 2018) [Kenya]**	To describe the provision of and access to inpatient neonatal services within Nairobi City County, and to explore access and evaluate readiness of public, private not-for-profit, and private-for-profit sector facilities	32 facilities in Nairobi City County that provide inpatient neonatal care	Delivery of quality care by the private sector	Cross-sectional	Mortality among the five largest facilities was 14.5% compared to 7.7% among the smaller facilities. Mortality was highest in the public sector (16.5%) compared with the mission (5.9%) and private (7.3%) sectors. Of the 1,296 deaths, 92% occurred in the 12/31 facilities with a structural score of 81–90%. Referral was reportedly more common from facilities with structural scores >90% (20% referral rate) and from those with structural scores 80% (32–34% referral rate) that were also often the smaller facilities.	Weak
**(Murphy, Gathara et al. 2018) [Kenya]**	To report on the quality of the process of care delivered to small and sick inpatient newborns across diverse facility settings in Nairobi	34 facilities in Nairobi City County that provide inpatient neonatal care	Delivery of quality care by the private sector	Retrospective review of medical records	Where recorded (n =1104), 90.6% were discharged alive, 7.2% died, 1.7% were referred and 0.5% absconded. Crude mortality, without adjustment for case-mix or acuity, was higher in public facilities (8.8% [95% CI: 6.5–11.7%]) compared with mission facilities (2.1% [95% CI: 0.3–12.3%]) and private-sector facilities (3.8% [95% CI: 2.2–6.5%]).	Weak

Author, year [country]	Aim / objective(s)	Setting and population	Intervention Description	Study design	Summary	Quality
**(Sharma, Powell-Jackson et al. 2017) [India]**	To describe and investigate the quality of care provided routinely, for uncomplicated labour and childbirth	Pregnant women with spontaneous, uncomplicated labour from 59 maternity facilities (29 from the private sector, 30 from the public sector)	Delivery of quality care by the private sector	Clinical observations	5 neonatal deaths in public facilities (218 deliveries observed) and 0 deaths in private facilities (64 deliveries observed)	Weak
**(Singh, Speizer et al. 2013) [Ghana]**	To evaluate the influence of the early phase of Project Fives Alive!, a national child survival improvement project, on key maternal and child health outcomes	Mothers, infants, and children under-five in 25 health centres and 2 hospitals that provide comprehensive emergency obstetric and neonatal care.	The intervention aimed to improve health outcomes in mothers, infants and children under-five by improving the coverage, quality, reliability and patient centeredness of the HIRD program across all public and faith-based facilities in Ghana	Interrupted time series	Neonatal mortality decreased from a mean of 2.5/1000 to 0.9/1000, and infant mortality decreased from a mean of 3.5/1000 to 2.3/1000 from the pre-intervention to post-intervention periods. Catholic facilities had a greater percentage of skilled deliveries but higher neonatal mortality.	Weak
**(Vora, Saiyed and Mavalankar 2018) [India]**	To determine the quality of free delivery care and examine the differences in quality of care between public sector facilities and accredited private sector facilities	1616 pregnant women of reproductive age	Delivery of quality care by the private sector	Descriptive analyses	Reported on live births; 98% of women had live births	Weak

Summary table of included studies reporting outcome data on child morbidity (n=14)

Author, year [country]	Aim / objective(s)	Setting and population	Intervention Description	Study design	Summary	Quality
**(Abuya, Molynuex et al. 2004) [Kenya]**	To describe the nature and practices of private health providers in rural Kenya, and user and health manager perspectives on quality of care offered	Private practitioners, private clinic users, and members of the district health management team in a rural area of Kenya	Delivery of quality care by the private sector	Mixed-methods	88 children (74%) were diagnosed to have clinical malaria	Weak
**(Angeles, Hutchinson and Khan 2003) [Bangladesh]**	To collect information on and monitor changes in USAID performance indicators, and to conduct an evaluation of the impact of the RSDP programme on the health of the catchment area population	Women ever married, aged 10-49 years: 9769 in the intervention areas, and 3176 in the comparison areas	Delivery of quality care by the private sector	Cohort analytic	Of the 6.2 percent of children with diarrhoea in the 2 weeks preceding the survey, most were treated with either Packet ORS or Labon gur solutions. The proportion receiving packet ORS increased from the Baseline Survey, as did the proportion receiving homemade water-salt-sugar/labon gur solutions. The overall proportion of children with diarrhoea receiving ORT (ORS and/or labon gur solution) increased from 62.9 percent in 1998 to 75.4 percent in 2001. A larger increase occurred in non-RSDP areas, from 50.9 percent to 67.5 percent of children with diarrhoea.<br><br>Just over 15 percent of children had symptoms of an acute respiratory infection in the 2 weeks preceding the survey, twice the observed rate in the Baseline Survey. In RSDP areas, 24 percent of children with ARI symptoms were taken to a health provider (excluding traditional doctors/pharmacies), considerably lower than the 32.4 percent who sought care at medical facilities in the Baseline Survey. In non-RSDP areas, the proportion seeking care was similar, 25 percent. The proportion seeking care from any source is however as high as 75 percent in both RSDP and non-RSDP areas. Among those who sought care from any source, less than 0.5 percent went to a RSDP provider.<br><br>Data show that 49.2 percent of children with diarrhoea in RSDP areas were not taken for treatment to a facility/provider. Of the remaining 50.8 percent for whom treatment was sought, almost	Moderate

Author, year [country]	Aim / objective(s)	Setting and population	Intervention Description	Study design	Summary	Quality
					three-fourths (74.8 percent) received treatment from the private medical sector, 16.7 percent from the public sector, and 3.5 percent received treatment at home. Only 2.3 percent were treated at RSDP facilities. This is nearly the same as the percentage who used RSDP facilities in the 1998 Baseline Survey. Among the private medical sector facilities, traditional doctors (17.4 percent) and pharmacies (12.6 percent) were the two main sources of diarrhoea treatment. Thana Health Complex (5.2 percent) was the most common public sector source for diarrhoea treatment. Of those who received treatment at home, most were treated by a non-medical person. In all divisions, the private medical sector and public sector facilities were the two main sources used for the treatment of childhood diarrhoea.	
**(Angeles, Lance et al. 2005) [Bangladesh]**	To collect information about knowledge, awareness, and use of services related to family planning, maternal and child health, provided by the project intervention	Women ever married, aged 10-49 years: 5691 in the intervention areas, and 4201 in the comparison areas	Delivery of quality care by the private sector	Cohort analytic	NSDP providers treated approximately 1% of children with diarrhoea, a share almost identical to that found in the 2001 survey. Private medical sector facilities were the most common source of treatment (at about 44%). The most popular of these facilities were private clinics/doctors (21.3%) and pharmacies (16.2%), followed by traditional doctors (6.7%).	Weak
**(Angeles, Lance and Khan 2006) [Bangladesh]**	To measure changes in the USAID performance indicators since the mid-project evaluation of the RSDP programme	7653 women from the intervention project areas and 4418 from the non-intervention project areas	Delivery of quality care by the private sector	Cohort analytic	40% of children with diarrhoea in project areas were taken for treatment to a facility/provider. This was seven percentage points lower than 2003. Of those who sought treatment, the vast majority did so from the private medical sector. Only 2.4% were treated at NSDP facilities. Among private medical sector facilities, traditional doctors (26.1%) and pharmacies (19.4%) were the two main providers. The 2001 and 2003 surveys reported similar patterns in the distribution of sources of diarrhoea treatment	Moderate
**(Annigeri, Prosser et al. 2004) [India]**	To provide suggestions for future public-private partnerships for USAID in India.	192 urban health centres in 74 municipalities in Andhra Pradesh	The Commissioner of Family Welfare built 192 urban health cantres. Intervention aimed at providing basic reproductive and	Qualitative interviews and field visits	NGOs reported significant reductions in childhood illnesses, 100 percent immunization rates, 100 percent institutional deliveries, improvements in child nutrition, and similar improvements in all other indicators that the health intervention intended to impact.	Weak

Author, year [country]	Aim / objective(s)	Setting and population	Intervention Description	Study design	Summary	Quality
			child health preventive care through: service delivery, community mobilisation, and behaviour change communication. Implemented via the local urban health centre and community outreach.			
**(Babirye, Engebretsen et al. 2014) [Uganda]**	To examine health care service and community-sub systems	821 child caretakers of children aged 10-23 months for survey, 58 women and 15 men for focus group discussions, 6 healthcare providers for key informant interviews	Delivery of quality care by the private sector	Mixed-methods	Respondents who received immunisation services from private facilities reported fever twice as commonly (OR 1.96, 95% CI 1.37-2.79) as those who received them from public facilities. This however did not remain significant at multivariable analysis. About 11% (95% CI 8-14%) of those whose children developed fever after immunisation did not seek care for the fever. Among those who did seek care, only 39% (95% CI 33-44%) sought it from health care workers. The rest used home remedies to treat the fever	Weak
**(Bangladesh. National Institute of Population, Training et al. 2016) [Bangladesh]**	To report the results of the 2014 Health Facility Survey	1548 health facilities	Delivery of quality care by the private sector	Quantitative findings from survey data	Each of the four priority medicines (ampicillin powder for injection, ceftriaxone powder for injection, gentamycin injection, and benzathine benzyl-penicillin injection) was available on the day of the survey in less than 10 percent of all facilities that offer curative care for sick children.<br><br>Just over half (69 percent excluding community clinics) of facilities that provide curative care services for sick children had some means for hand cleaning.<br><br>The majority of facilities have the five basic medicines: mebendazole/albendazole (88 percent), paracetamol	Moderate

Author, year [country]	Aim / objective(s)	Setting and population	Intervention Description	Study design	Summary	Quality
					syrup/suspension (86 percent), amoxicillin syrup/suspension/dispersible (81 percent), ORS (79 percent), and zinc tablets or syrup (66 percent).<br><br>9 percent (8 percent excluding community clinics) of facilities that offer child curative care have all of the 10 items considered by WHO as necessary for a facility to be ready to provide child curative care.	
**(Carter, Ndhlovu et al. 2016) [Zambia]**	To assess the feasibility of collecting geographically and temporally concurrent household and health care provider data	355 rural and 469 urban households with at least one eligible mother of a child under 5 in Choma District, Southern Province, Zambia	Delivery of quality care by the private sector	Descriptive statistics and facility evaluations	Among the 1084 children included in the household survey, 35% of urban children and 36% of rural children experienced at least one illness meeting DHS criteria in the 2 weeks preceding the survey. Fever was the most commonly experienced symptom in both the rural and urban areas. Mothers reported care was sought for 79% of rural children and 67% of urban children with an illness.<br><br>Government health centres were the primary reported source of care in both the urban (60%) and rural (61%) areas. In the rural area, 18% of children were taken to a CBA for care. A small number of children were taken to shops and traditional practitioners in the rural area. In the urban area, care was sought for 5% of children from informal shops. Hospitals, pharmacies, and private facilities accounted for a small number of care-seeking events in the urban area.	Strong
**(Danel and Forgia 2005) [Guatemala]**	To assess the performance of Guatemala's programme to extend coverage of basic health services, and determine the relative economic efficiency of NGO and Ministry of Health and Social Protection providers in delivering basic health services	161 signed agreements between NGOs and the government, 88 under contract, estimated coverage of 3,200,000 people	Delivery of quality care by the private sector	Regression analyses	Women in mixed provider communities were more likely to have used ORS [oral rehydration] during their child's last episode of diarrhoea and more likely to say they knew how to use ORS. Compared to traditional provider communities, women in direct provider communities were slightly more likely to have used ORS during their child's last episode of diarrhoea and to say they knew how to use it.<br><br>The percentage of women with children under two years of age who had ORS in the home was low (less than 20 percent) in all three types of communities. Use of medical care for diarrhoea and respiratory diseases in children was high in all three groups	Weak

Author, year [country]	Aim / objective(s)	Setting and population	Intervention Description	Study design	Summary	Quality
					of communities, varying from 46 percent to 54 percent for diarrhoea and from 68 percent to 77 percent for respiratory illness. Children in mixed provider and traditional communities were more likely to have received medical care during an episode of illness than children in direct provider communities. Women were more likely to seek medical care for infants with respiratory dis- ease than for those with diarrhoea. More than two-thirds of mothers sought care for infants and children with respiratory illness	
**(De Savigny, Mayombana et al. 2004) [Tanzania]**	To anlyse care-seeking events in malaria death records through longitduninal demographic surveillance	Monitor of 85,000 people in 17,000 households across 32 villages in Rufiji District	Delivery of quality care by the private sector	Mixed-methods: quantitative surveillance data and qualitative semi-structured interviews, focus group discussions and case histories	The initial treatment-seeking choice for children with acute febrile illness who were less than five years of age was modern care (78.7%), whereas only 9.4% used traditional care initially. The remainder (11.9%) sought no care.	Weak
**(Health Partners International and Montrose 2014) [Uganda]**	The study had three objectives:<br>1. To compare differences in the use of partographs between the RBF and IBF health facilities and assess whether these are associated with perinatal outcomes.<br>2. To compare differences in the use of partographs	31 faith-based, private not-for-profit health providers in Acholi sub region and ten in control area in Lango sub region	Northern Uganda Health Programme (NU Health 2011-2015) aimed to strengthen local and national mechanisms for governance and accountability and improve access to healthcare through	Cohort analytic and regression analyses	When comparing the RBF and IBF regions, correct malaria treatment was more likely to be provided in the RBF region at both baseline and after one year, although there was little change in the proportion of children treated correctly within the RBF group. After adjusting the OR for facility level, sex and age of the child, there was no significant difference in the changes in the two regions between the baseline (OR 0.84 CI 0.56-1.27) and first year of implementation (OR 1.33 CI 0.59-1.32).<br><br>When comparing the RBF and IBF regions, the RBF facilities performed much better than the IBF facilities (see Fig 11 below).	Weak

Author, year [country]	Aim / objective(s)	Setting and population	Intervention Description	Study design	Summary	Quality
	between the RBF and IBF health facilities and assess whether these are associated with occurrence of emergency Caesarean Sections<br>3. To compare differences in prescription practices between the RBF and IBF health facilities for common childhood illnesses (malaria, pneumonia and diarrhoea) and assess the overall management of sick children.		the use of results-based financing.		In the baseline year, when the OR was adjusted for facility level, and sex and age of the child, the odds of a case being treated correctly in the RBF region were almost twice that of the IBF region (OR 1.84 CI 1.07-3.20). In year one, although both regions showed improvement, the adjusted odds of a case being treated correctly in the RBF region were almost six times that in the IBF region (OR 5.66 CI 3.70-8.66)	
**(Mohanan, Babiarz et al. 2016) [India]**	To evalute the imapct of the World Health Partners Sky programme	36,315 children under give in twelve districts across the state of Bihar in 2011; 31,635 in 2014	SkyHealth telemedical facilities were established in villages with internet connectivity, to allow patient consultations and remote assessments of certain conditions. Providers who have facilities with telemedicine technology were franchised alongside partnerships with SkyCare providers	Case-Control	Found no significant effect of the WHP-Sky program on population health outcomes. Estimated program effects on the prevalence of diarrhoea (a 1.5-percentage-point change) and the prevalence of pneumonia (a 0.1-percentage-point) were statistically indistinguishable from zero	Weak

Author, year [country]	Aim / objective(s)	Setting and population	Intervention Description	Study design	Summary	Quality
			and rural healthcare providers. All received training on basic service delivering, access to marketing services, and a predictable supply of brand-name adequate-quality drugs			
**(Sidharthan, Ganapathy et al. 2015) [India]**	To evidence treatment and supportive care approaches for better health outcomes	76 patients above one year and less than 14 years	Delivery of quality care by the private sector	Analysis of clinic informati on	One patient (1.3%) died during induction, 1 was censored at loss to follow-up upon transfer of treatment to another centre, none abandoned treatment, and 6 (7.9%) relapsed, with 1 (1.3%) relapse within 90 days of diagnosis	Weak
**(Wallen, Blenden et al. 2017) [Various (23 countries)]**	To document the experience of outcomes of the International Children's Heart Foundation	3784 children seeking heart related care	International Children's Heart Foundation team of 15 to 30 medical personnel volunteering at local healthcare institutions, to collaborate on patient care, education, and training.	Regressio n analyses	The average patient age was 5 years with a range from 4 days to 60.6 years. Fifty-one percent was male with 20% presenting with some degree of malnourishment, with 4% being emaciated. Nutritional status was determined by clinical evaluation of a multidisciplinary team. Twenty seven percent required preoperative intensive care unit (ICU) care and 11% required reoperation. Most patients requiring preoperative ICU care required hydration with close fluid balance monitoring.	Weak

Summary table of included studies reporting outcome data on child mortality (n=10)

Author, year [country]	Aim / objective(s)	Setting and population	Intervention Description	Study design	Summary	Quality
**(Angeles, Hutchinson and Khan 2003) [Bangladesh]**	To collect information on and monitor changes in USAID performance indicators, and to conduct an evaluation of the impact of the RSDP programme on the health of the catchment area population	Women ever married, aged 10-49 years: 9769 in the intervention areas, and 3176 in the comparison areas	Delivery of quality care by the private sector	Cohort analytic	The infant mortality rate in RSDP areas for the 5-year period preceding the survey was 77 deaths per 1,000 live births, while the child mortality rate was 28.6 deaths per 1,000. These rates are higher than the rates in non-RSDP areas – 70.5 and 24.1 deaths per 1,000 live births respectively	Moderate
**(Angeles, Lance et al. 2005) [Bangladesh]**	To collect information about knowledge, awareness, and use of services related to family planning, maternal and child health, provided by the project intervention	Women ever married, aged 10-49 years: 5691 in the intervention areas, and 4201 in the comparison areas	Delivery of quality care by the private sector	Cohort analytic	The child mortality rate fell slightly, from 21.1 to 12.6 deaths per 1,000 live births. In non-NSDP areas, mortality (particularly infant) fell by a larger margin	Weak
**(Angeles, Lance and Khan 2006) [Bangladesh]**	To measure changes in the USAID performance indicators since the mid-project evaluation of the RSDP programme	7654 women from the intervention project areas and 4418 from the non-intervention project areas	Delivery of quality care by the private sector	Cohort analytic	The child mortality rate was 18.7 deaths per 1,000, down from roughly 20 per 1,000 in 2003, and 28.6 in 2001. The overall child mortality rate in project areas was 19.1 for the full sample and 26 for the poorest. In both NSDP and non-NSDP areas, mortality rates have declined significantly over the past 15 years.	Moderate
**(Bjorkman-Nykvist, Guariso et al. 2015) [Uganda]**	To asses the impact of non-profit entrepreneurial models of community health delivery	8120 households in 214 villages across 10 districts	A cluster-randomized controlled trial from 214 rural villages in 10 districts in Uganda. In treatment villages, Living Goods and BRAC Community Health Promoters conducting home visits, educating households on essential health	Randomized controlled trial	The intervention reduced under-five mortality rate by 25%.	Weak

Author, year [country]	Aim / objective(s)	Setting and population	Intervention Description	Study design	Summary	Quality
			behaviours and selling preventive and curative health products at 20-30% below prevailing retail prices were deployed over a three-year period (2011- 2013)			
**(Bojalil, Kirkwood et al. 2007) [Mexico]**	To provide information to better implement interventions linked with the Integrated management of Childhood Illnesses programme	75 mothers whose child (up to age 5) had died within the last 90 days	Delivery of quality care by the private sector	Analysis of "Death narratives" and competency evaluations	Case management practices by doctors were linked to 27 (36%) of the deaths. In each case, the severity of the disease had not been recognized by the doctor, resulting in inadequate treatment, including failure to prescribe ORS for diarrhoea.	Weak
**(Damonti, Doykos et al. 2012) [Botswana, Lesotho, Swaziland, Malawi, Uganda, Tanzania]**	To establish, sustain, and expand the provision of high-quality HIV treatment and care for children	103,731 children	The establishment of Children's Clinical Centers of Excellent in Botswana, Lesotho, Swaziland, Uganda, Tanzania and Malawi, which were equipped to provide emergency care for acutely ill children, outpatient care for children living with and exposed to HIV, a pharmacy, key laboratory services, voluntary counselling and testing, psychosocial care, and training for local and visiting	Descriptive statistics	They found that the overall materality rate of 3.35 deaths per 100-patient years compared favourably to comparable studies.	Weak

Author, year [country]	Aim / objective(s)	Setting and population	Intervention Description	Study design	Summary	Quality
			healthcare professionals.			
**(Greve and Schattan Ruas Pereira Coelho 2017) [Brazil]**	To explore the impact of contracting out not-for-profit organisations in the state of São Paulo	Employees on external contracts working in primary health care in 206 municipalities in the state of São Paulo	Intervention contracted pre-certified non-profit or non-governmental organizations to take part in the delivery of health care services	Case-Control	Municipalities implementing external contracts in the primary health care sector had lower infant and child mortality rates and lower hospitalization rates for preventable and respiratory diseases. Parameter estimates indicate that contracting out reduced child mortality and hospitalisation but it could not be rejected that these parameter estimates are zero.	Weak
**(J-PAL Policy Briefcase 2019) [Uganda]**	To evaluate the Community Health Promoter program by BRAC Uganda	Community health promotors in 214 villages	Comprehensive training on health education and business skills for community health promotors, in order to provide home visits, health education, basic medical advice and treatment, and referrals to nearby clinics, as well as selling health-related commodities.	Randomized controlled trial	Child mortality dropped by 27 percent in CHP villages relative to the comparison group	Weak
**(Kohnke, Mukherjee and Sinha 2017) [China]**	To understand how international NPOs enable the long-term delivery of surgical care in underserved communities	15 key individuals from Children's Heartlink and partner organisations; data from 11 observation years	Children's Heart-Link intervention provides education, technical support, diagnostic and treatment capabilities, and medical equipment to parnter organisations, as well as skill development and resources for medical staff.	Mixed-methods	The in-hospital mortality rate was 5.3% for this sample of patients. This was higher than the normal for the intervention facility, but was reported to be quite low according to the Children’s Heartlink mission team members familiar with the site	Weak

Author, year [country]	Aim / objective(s)	Setting and population	Intervention Description	Study design	Summary	Quality
**(Wallen, Blenden et al. 2017) [Various (23 countries)]**	To document the experience of outcomes of the International Children's Heart Foundation	3783 children seeking heart related care	International Children's Heart Foundation team of 15 to 30 medical personnel volunteering at local healthcare institutions, to collaborate on patient care, education, and training.	Regression analyses	In-hospital mortality was 8.1%. The reoperation rate was 11.1%, of which 5.7% of the total cohort returned to the operating room for a bleeding indication.	Weak

Abdella, A., T. Fetters, J. Benson, E. Pearson, Y. Gebrehiwot, K. Andersen, H. Gebreselassie and S. Tesfaye (2013). "Meeting the need for safe abortion care in Ethiopia: Results of a national assessment in 2008." Global Public Health **8**(4): 417-434.

Abuya, T. O., C. S. Molynuex, A. S. Orago, S. Were and V. Marsh (2004). "Quality of care provided to febrile children presenting in rural private clinics on the Kenyan coast." African health sciences **4**(3): 160-170.

Adisasmita, A., P. E. Deviany, F. Nandiaty, C. Stanton and C. Ronsmans (2008). "Obstetric near miss and deaths in public and private hospitals in Indonesia." BMC Pregnancy and Childbirth **8**: 10.

Al Haque, N., M. E. Chowdhury, N. L. Huq, A. Ahmed, S. K. DasGupta and M. A. Quaiyum (2010). Baseline findings: Shahjadpur Integrated Maternal and Neonatal Health Project, Dhaka, Bangladesh, International Centre for Diarrhoeal Disease Research, Bangladesh [ICDDR,B], 2010 Dec.**:** [82] p.

Allam, R. R., G. Oruganti, C. Uthappa, N. Simhachalam, J. Rajesh and V. Yeldandi (2016). "APAIDSON program evaluation of the largest private public partnership consortium for HIV/AIDS care and treatment in India." International Journal of Infectious Diseases **45**(SUPPL. 1): 215.

Aman, H., S. Negash and L. Yusuf (2014). "Cesarean delivery practices in teaching public and non-government / private MCH hospitals, Addis Ababa." Ethiopian Journal of Health Development **28**(1): 22-28.

Angeles, G., P. Hutchinson and M. S. Khan (2003). 2001 Rural Service Delivery Partnership Evaluation Survey. Household survey report, Chapel Hill, North Carolina, University of North Carolina at Chapel Hill, Carolina Population Center [CPC], MEASURE Evaluation, 2003 Feb.**:** [182] p.

Angeles, G., P. Lance, P. Hutchinson, S. N. Mitra and S. Islam (2005). 2003 Urban NGO Service Delivery Program (NSDP) evaluation survey, [Chapel Hill, North Carolina], University of North Carolina at Chapel Hill, Carolina Population Center [CPC], MEASURE Evaluation, 2005 Mar.**:** [193] p.

Angeles, G., P. Lance and M. S. Khan (2006). 2005 Rural NGO Service Delivery Program (NSDP) evaluation survey, Chapel Hill, North Carolina, University of North Carolina at Chapel Hill, Carolina Population Center [CPC], MEASURE Evaluation, 2006 Aug.**:** [186] p.

Annigeri, V. B., L. Prosser, J. Reynolds and R. Roy (2004). An assessment of public-private partnership opportunities in India, Washington, D.C., LTG Associates, Population Technical Assistance Project [POPTECH], 2004 Nov.**:** [71] p.

Anwar, I., T. Begum, A. Rahman, H. Nababan and R. Islam (2016). "Quality of Maternal and Neonatal Health (MNH) care in for profit private sectors in urban Bangladesh." European Journal of Public Health **26**: 264-264.

Audinarayana, N. (2008). "Are government health facilities losing their charm in extending maternal and child health care services? A critical review of studies from Tamil Nadu." Health and Population: Perspectives and Issues **31**(4): 234-246.

Babirye, J. N., I. M. Engebretsen, E. Rutebemberwa, J. Kiguli and F. Nuwaha (2014). "Urban settings do not ensure access to services: findings from the immunisation programme in Kampala Uganda." BMC Health Serv Res **14**: 111.

Bangladesh. National Institute of Population, R., Training, H. Bangladesh. Ministry of, W. Family, C. Associates for, R. Population and I. C. F. I. D. Program (2016). Bangladesh Health Facility Survey 2014. Final report, Dhaka, Bangladesh, NIPORT, 2015 Apr.**:** 276 p.

Bhargava, A., S. Chowdhury and K. K. Singh (2005). "Healthcare infrastructure, contraceptive use and infant mortality in Uttar Pradesh, India." Economics & Human Biology **3**(3): 388-404.

Bjorkman-Nykvist, M., A. Guariso, J. Svensson and D. Yanagizawa-Drott. (2015). "Abstract: Evaluating the impact of the Living Goods entrepreneurial model of community health delivery in Uganda: A cluster-randomized controlled trial." Retrieved 4 Feb 2020, from https://healthmarketinnovations.org/sites/default/les/Abstract_CHP2014.pdf.

Bojalil, R., B. R. Kirkwood, M. Bobak and H. Guiscafre (2007). "The relative contribution of case management and inadequate care-seeking behaviour to childhood deaths from diarrhoea and acute respiratory infections in Hidalgo, Mexico." Tropical Medicine and International Health **12**(12): 1545-1552.

Carter, E., M. Ndhlovu, E. Nkhama, M. Munos, J. Katz and T. P. Eisele (2016). "Linking household and point-of-care data to estimate coverage of appropriate management of childhood illness in Southern Province, Zambia." American Journal of Tropical Medicine and Hygiene **95**(5 Supplement 1): 262-263.

Damonti, J., P. Doykos, R. S. Wanless and M. Kline (2012). "HIV/AIDS in African children: the Bristol-Myers Squibb Foundation and Baylor response." Health Aff (Millwood) **31**(7): 1636-1642.

Danel, I. and G. Forgia (2005). "Contracting for basic health care in rural Guatemala - Comparison of the performance of three delivery models." Health Systems Innovations in Central America: Lessons and Impact of New Approaches: 49-88.

De Savigny, D., C. Mayombana, E. Mwageni, H. Masanja, A. Minhaj, Y. Mkilindi, C. Mbuya, H. Kasale and G. Reid (2004). "Care-seeking patterns for fatal malaria in Tanzania." Malaria Journal **3**: 27.

Greve, J. and V. Schattan Ruas Pereira Coelho (2017). "Evaluating the impact of contracting out basic health care services in the state of São Paulo, Brazil." Health Policy Plan **32**(7): 923-933.

Health Partners International and Montrose (2014). NU Health. Clinical audit of maternal and child health care services in the context of results based financing in Northern Uganda. Kampala (Uganda), NU Health Programme**:** 44 p.

Huda, F. A., A. Ahmed, E. R. Ford and H. B. Johnston (2015). "Strengthening health systems capacity to monitor and evaluate programmes targeted at reducing abortion-related maternal mortality in Jessore district, Bangladesh." BMC health services research **15**: 426.

J-PAL Policy Briefcase (2019). in the business of saving lives. Cambridge, MA, USA, Abdul Latif Jameel Poverty Action Lab.

Jenkins, K. J., A. R. Castaneda, K. M. Cherian, C. A. Couser, E. K. Dale, K. Gauvreau, P. A. Hickey, J. K. Kupiec, D. F. Morrow, W. M. Novick, S. J. Rangel, B. Zheleva and J. T. Christenson (2014). "Reducing Mortality and Infections After Congenital Heart Surgery in the Developing World." Pediatrics **134**(5): E1422-E1430.

Karki, C., M. Ojha and R. T. Rayamajhi (2009). "Baseline survey on functioning of abortion services in government approved CAC centers in three pilot districts of Nepal." Kathmandu Univ Med J (KUMJ) **7**(25): 31-39.

Kohnke, E. J., U. K. Mukherjee and K. K. Sinha (2017). "Delivering Long-Term Surgical Care in Underserved Communities: The Enabling Role of International NPOs as Partners." Production and Operations Management **26**(6): 1092-1119.

Kumutha, J., G. V. R. Rao, B. N. Sridhar and D. Vidyasagar (2015). "The GVK EMRI maternal and neonatal transport system in India: a mega plan for a mammoth problem." Seminars in Fetal & Neonatal Medicine **20**(5): 326-334.

Maru, D., S. Maru, I. Nirola, J. Gonzalez-Smith, A. Thoumi, P. Nepal, P. Chaudary, I. Basnett, K. Udayakumar and M. McClellan (2017). "Accountable Care Reforms Improve Women's And Children's Health In Nepal." Health Affairs **36**(11): 1965-1972.

McIntosh, N., A. Grabowski, B. Jack, E. L. Nkabane-Nkholongo and T. Vian (2015). "GLOBAL. A Public-Private Partnership Improves Clinical Performance In A Hospital Network In Lesotho." Health Affairs **34**(6): 954-962.

Mendez, E. and Associates (2014). End-of-project performance evaluation of USAID / Caucasus Sustaining Family Planning and Maternal and Child Health (SUSTAIN) project in Georgia. Final report, Bethesda, Maryland, Mendez, England and Associates, 2014 Dec 12.**:** [91] p.

Mohanan, M., K. S. Babiarz, J. D. Goldhaber-Fiebert, G. Miller and M. Vera-Hernández (2016). "Effect Of A Large-Scale Social Franchising And Telemedicine Program On Childhood Diarrhea And Pneumonia Outcomes In India." Health Aff (Millwood) **35**(10): 1800-1809.

Murphy, G. A. V., D. Gathara, N. Abuya, J. Mwachiro, S. Ochola, R. Ayisi and M. English (2018). "What capacity exists to provide essential inpatient care to small and sick newborns in a high mortality urban setting? - A cross-sectional study in Nairobi City County, Kenya." PLoS One **13**(4): e0196585.

Murphy, G. A. V., D. Gathara, J. Mwachiro, N. Abuya, J. Aluvaala, M. English, S. Ochola, R. Ayisi, A. Wasunna, F. Were, C. Mutinda, B. Maina, C. Mutiso, D. Githanga, D. Kimutai, R. Musoke, R. Ochieng, W. Macharia, R. Nyamai and G. on behalf of the Health Services that Deliver for Newborns Expert (2018). "Effective coverage of essential inpatient care for small and sick newborns in a high mortality urban setting: a cross-sectional study in Nairobi City County, Kenya." BMC Medicine **16**(1): 72.

Nelson, D., M. Corbett, F. Githiori, R. Mason Jr, P. Muhuhu, R. Mulindi and F. Yumkella (2002). The right provider for the right place: private nurse-midwives offering primary-level post-abortion care in Kenya. PRIME II Dispatch. **4**.

Ogu, R., F. Okonofua, A. Hammed, E. Okpokunu, A. Mairiga, A. Bako, T. Abass, D. Garba, A. Alani and K. Agholor (2012). "Outcome of an intervention to improve the quality of private sector provision of postabortion care in northern Nigeria." International Journal of Gynecology & Obstetrics **118**: S121-126.

Rahman, M., U. Rob and T. Kibria (2009). Implementation of maternal health financial scheme in rural Bangladesh.

Ramachandar, L. and P. J. Pelto (2002). "The role of village health nurses in mediating abortions in rural Tamil Nadu, India." Reprod Health Matters **10**(19): 64-75.

Ramachandar, L. and P. J. Pelto (2004). "Abortion Providers and Safety of Abortion: A Community-Based Study in a Rural District of Tamil Nadu, India." Reproductive Health Matters **12**(24, Supplement): 138-146.

Save the Children (2007). Umoyo Newtork Capacity Building for Quality HIV / AIDS Services Project: Final Report, Save the Children.

Sharan, M., S. Ahmed, A. Malata and K. Rogo (2012). "The quality of public and private maternal health systems in malawi [NOTE: just an abstract of conference presentation - no full PDF]." International Journal of Gynecology and Obstetrics **119**(SUPPL. 3): S479-S480.

Sharma, G., T. Powell-Jackson, K. Haldar, J. Bradley and V. Filippi (2017). "Quality of routine essential care during childbirth: clinical observations of uncomplicated births in Uttar Pradesh, India." Bull World Health Organ **95**(6): 419-429.

Sidharthan, N., R. Ganapathy, P. Gangadharan, S. Soman, K. Pavithran, R. Prabhu, H. V. Raju, A. Rajan, P. Boyella, R. Arun, S. C. Howard and A. Pillai (2015). "Strict treatment regimen and febrile neutropenia guidelines allow favorable outcomes for pediatric all in Southern India." Blood **126**(23): 4510.

Singh, K., I. Speizer, S. Handa, R. O. Boadu, S. Atinbire, P. M. Barker and N. A. Twum-Danso (2013). "Impact evaluation of a quality improvement intervention on maternal and child health outcomes in Northern Ghana: early assessment of a national scale-up project." Int J Qual Health Care **25**(5): 477-487.

Vora, K. S., S. L. Saiyed and D. V. Mavalankar (2018). "Quality of Free Delivery Care among Poor Mothers in Gujarat, India: A Community-Based Study." Indian Journal of Community Medicine **43**(3): 224-228.

Wallen, T., R. Blenden, I. Zafurallah, T. Vesel and R. Soto (2017). "Programmatic changes to reduce mortality and morbidity in humanitarian congenital heart surgery." Cardiology in the Young **27**(4): S227-S228.
