# Supplementary material for: Impact of Private Sector Delivery of Quality Care on Maternal, Newborn, and Child Health Outcomes in Low- and Middle-Income Countries: A Systematic Review
Source: Ann Glob Health. 2025 Jun 20;91(1):35. doi: 10.5334/aogh.4596 (PMC12180434; doi:10.5334/aogh.4596)
Supplement: Supplementary Annex 2. — Intervention characteristics among studies reporting specific interventions that went beyond the generic delivery of quality care. [file agh-91-1-4596-s2.pdf]

Supplementary Annex 2: Intervention characteristics amongst studies reporting specific interventions that went beyond the generic delivery of quality care

Characteristics	*Number of specific intervention studies included in final inventory (%)*	*Number of studies examining morbidity and/or mortality among mothers, newborns, and children (%)*
Intervention quantity		
Single intervention	42 (72.4%)	15 (71.4%)
Multiple interventions	16 (27.6%)	6 (28.57%)
Focus on supply-side or demand-side factors		
Supply-side	37 (63.8%)	12 (57.1%)
Demand-side	2 (3.5%)	--
Both	18 (31.0%)	9 (42.9%)
Unclear	1 (1.7%)	--
Intervention type: on-site support for QI		
No	27 (46.6%)	14 (66.7%)
Yes	31 (53.4%)	7 (33.3%)
Intervention type: data systems		
No	43 (74.1%)	15 (71.4%)
Yes	15 (25.9%)	6 (28.6%)
Intervention type: learning systems		
No	29 (50.0%)	8 (38.1%)
Yes	29 (50.0%)	13 (61.9%)
Intervention type: program management		
No	29 (50.0%)	12 (57.1%)
Yes	29 (50.0%)	9 (42.9%)
Intervention type: advocacy		
No	31 (53.4%)	11 (52.4%)
Yes	27 (46.6%)	10 (47.6%)
Intervention type: policy and strategy development		
No	46 (79.3%)	17 (80.9%)
Yes	12 (20.7%)	4 (19.1%)
Intervention recipient: women during pregnancy, childbirth, and/or postpartum		
Indirect recipient	16 (11.5%)	9 (17.7%)
Direct recipient	47 (33.8%)	12 (23.5%)
Not a recipient	76 (54.7%)	30 (58.8%)
Intervention recipient: Subgroup of women during pregnancy, childbirth, and/or postpartum		
Indirect recipient	4 (2.9%)	4 (7.8%)
Direct recipient	2 (1.4%)	1 (2.0%)
Not a recipient	133 (95.7%)	46 (90.2%)
Intervention recipient: women defined more generally		
Indirect recipient	12 (8.6%)	6 (11.8%)
Direct recipient	12 (8.6%)	8 (15.7%)

Characteristics	*Number of specific intervention studies included in final inventory (%)*	*Number of studies examining morbidity and/or mortality among mothers, newborns, and children (%)*
Not a recipient	115 (82.7%)	37 (72.6%)
Intervention recipient: newborns		
Indirect recipient	15 (10.8%)	8 (15.7%)
Direct recipient	14 (10.1%)	7 (13.7%)
Not a recipient	110 (79.1%)	36 (70.6%)
Intervention recipient: children		
Indirect recipient	15 (10.8%)	8 (15.7%)
Direct recipient	18 (13.0%)	11 (21.6%)
Not a recipient	106 (76.3%)	32 (62.8%)
Intervention recipient: health care providers		
Indirect recipient	6 (4.3%)	5 (9.8%)
Direct recipient	84 (60.4%)	31 (70.8%)
Not a recipient	49 (35.3%)	15 (29.4%)
Type of intervention evaluation		
Impact	36 (45.6%)	9 (45.0%)
Process	12 (21.1%)	5 (25.0%)
Impact and process	15 (26.3%)	4 (20.0%)
Multiple (e.g., impact, process, economic)	4 (7.0%)	2 (10.0%)
