# Supplementary material for: Impact of Private Sector Delivery of Quality Care on Maternal, Newborn, and Child Health Outcomes in Low- and Middle-Income Countries: A Systematic Review
Source: Ann Glob Health. 2025 Jun 20;91(1):35. doi: 10.5334/aogh.4596 (PMC12180434; doi:10.5334/aogh.4596)
Supplement: Supplementary Annex 3. — Summary table of included studies reporting outcome data on infant and child growth (n = 9). [file agh-91-1-4596-s3.pdf]

Supplementary Annex 3: Summary table of included studies reporting outcome data on infant and child growth (n=9)

Author, year [country]	Aim / objective(s)	Setting and population	Intervention Description	Study design	Summary	Quality
**(Alireza, Farahbakhsh et al. 2006) [Iran]**	To evaluate a new model of child and maternity health services	9 co-operative health centres and 18 public health centres	Establishment of co-operative health centres. Services include vaccination, MCH care, family planning, environmental health, and outpatient visits.	Case-control	There were no significant differences in children's growth percentiles between co-operate and public health centres.	Weak
**(Aman, Negash and Yusuf 2014) [Ethiopia]**	To compare caesarean delivery practices between teaching government and non-government and private fee-for-service MCH hospitals	944 medical charts (479 from government hospitals and 465 from non-government hospitals)	Delivery of quality care by the private sector	Case study analysis of associations	No significant difference was observed in the proportion of weight between 2500 gm – 3999 gm in both groups, 362 (78.5%) vs. 381 (81%), in teaching hospitals and non-governmental hospitals but the proportion of low birth weight and very low birth weight were significantly different in the teaching hospitals compared to non-governmental hospitals, 117 (22.1%) vs. 49 (10.1%) and 16 (3.1) vs. 3 (0.6%)	Weak
**(Arrieta, García-Prado and Guillén 2011) [Bolivia, Colombia, Dominican Republic, Guatemala, Nicaragua, Peru]**	To compare the effectiveness of private and public sector prenatal care	Demographic and Health Surveys	Delivery of quality care by the private sector	Regression analyses	In general, for public and private facilities, over-utilization of prenatal services has a limited impact on birth weights. It is associated to an increase of 1.6% in normal weight and a decrease of 1.7% in low birth weight, but only for countries in group I. In all other cases, it is not statistically significant. However, under-utilization does have a strong and significant impact on weight. The coefficient for low number of prenatal visits is statistically significant for any measure of birth weight in group I. This finding indicates that if the number of prenatal visits is lower than the benchmark, newborn weight could be around 3–4% lower, independently of the type of facility ownership (public or private)	Strong
**(Bangladesh. National Institute of**	To report the results of the 2014 Health Facility Survey	1549 health facilities	Delivery of quality care by the private sector	Quantitative findings	Child growth monitoring is not as available as curative care services in health facilities in Bangladesh. Only 62 percent (65 percent excluding community clinics) of facilities offer the service. Among the facilities	Moderate

Author, year [country]	Aim / objective(s)	Setting and populatio n	Intervention Description	Study design	Summary	Quality
**Population, Training et al. 2016) [Bangladesh]**				from survey data	that offer child growth monitoring, 84 percent provide the service during all working days in a week (not shown in table). Within the variation in the availability of growth monitoring services by facility type, the majority of (55 to 78 percent) of public and NGO facilities offer growth monitoring service for children, as compared with only two in ten private hospitals. Urban facilities (55 percent) are less likely to offer the service than rural facilities (63 percent); this is probably because of the high density of private facilities in urban areas.	
**(Danel and Forgia 2005) [Guatemala]**	To assess the performance of Guatemala's programme to extend coverage of basic health services, and determine the relative economic efficiency of NGO and Ministry of Health and Social Protection providers in delivering basic health services	162 signed agreements between NGOs and the government, 88 under contract, estimated coverage of 3,200,000 people	Delivery of quality care by the private sector	Regression analyses	A minority of children under two in each of the three groups had received growth monitoring in the three months prior to the survey (3–12%). Among those who did, nearly all were weighed, though children from traditional and direct provider communities were more likely to be weighed than those in mixed provider communities (93% and 100% compared to 86%). Children in direct provider communities were more likely to have their weight noted on their carnet. However, fewer children had their height measured during their last checkup (68–81%).	Weak
**(Farahbakhsh , Sadeghi-Bazargani et al. 2012) [Iran]**	To conduct a comparative observation study of health service delivery programmes over the period 2001-2002	1000 households, 20 clients per health facility, all physicians and professional staff in the health facilities	Delivery of quality care by the private sector	Comparative observationa l study with regression analyses	The sole index found to have better status in primary health centres was the percentage of children having a growth chart. The figure was equal to 67.5% in cooperative health centres and 83.5% in primary health centres ($P<0.05$). Child growth sheets were filled in accurately in 69.4% of cooperative health centres and 59% of primary health centres. Growth status of children based on growth percentiles was reported as favourable in 89.8% of cooperative health centres and 74.3% of primary health centres.	Weak
**(Khan, Owais et al. 2017) [Pakistan]**	To explore how intended intervention components were implemented and experienced by private	16 intervention clinics and 16 control clinics	Early childhood development care intervention using a structured clinic-based, quarterly	Mixed-methods	The provision of infantometers and weighing machines for active child growth monitoring in the trial created a ripple effect at these clinics, as other mothers (not registered in the trial) also started requesting for their children to be measured; this indicated a general interest of mothers to know more about their child’s wellbeing. Recording of child	Weak

Author, year [country]	Aim / objective(s)	Setting and populatio n	Intervention Description	Study design	Summary	Quality
	care providers and clients, and to identify how intervention components could be further adapted for scaling up care	in Lahore and Rawalpindi	tool-assisted counselling session for mother-child pairs. Each intervention arm offered each mother a 10-minute counselling session when her child was <6 weeks old, again at 3 months, 6 months, and 9 months. The intervention also trained private doctors and clinical assistants.		development milestones was not a requirement of ECD care delivery in the trial. Therefore, it was not possible to assess the providers' adherence to care protocols and child referrals.	
**(Singh, Speizer et al. 2013) [Ghana]**	To evaluate the influence of the early phase of Project Fives Alive!, a national child survival improvement project, on key maternal and child health outcomes	Mothers, infants, and children under-five in 25 health centres and 2 hospitlas that provide comprehensiv e emergency obstetric and neonatal care.	The intervention aimed to improve health outcomes in mothers, infants and children under-five by improving the coverage, quality, reliability and patient centeredness of the HIRD program across all public and faith-based facilities in Ghana	Interrupted time series	Hospitals, which often have the sickest patients, had higher mortality and a higher percentage of underweight infants attending child wellness clinics than health centers.	Weak
**(Wallen, Blenden et al. 2017)**	To document the experience of outcomes of the International	3785 children seeking heart related care	International Children's Heart Foundation team of 15 to 30 medical	Regression analyses	The average patient age was 5 years with a range from 4 days to 60.6 years. Fifty-one percent was male with 20% presenting with some degree of malnourishment, with 4% being emaciated. Nutritional status was determined by clinical evaluation of a multidisciplinary team.	Weak

Author, year [country]	Aim / objective(s)	Setting and populatio n	Intervention Description	Study design	Summary	Quality
**[Various (23 countries)]**	Children's Heart Foundation		personnel volunteering at local healthcare institutions, to collaborate on patient care, education, and training.			

Alireza, N., M. Farahbakhsh, K. Ashjaei, T. Djafarsadegh, H. Sadeghi-Bazargani and Z. Akram (2006). "Maternity and Child Health Care Services Delivered by Public Health Centers Compared to Health Cooperatives: Iran`s Experience." Journal of Medical Sciences **6**.

Aman, H., S. Negash and L. Yusuf (2014). "Cesarean delivery practices in teaching public and non-government / private MCH hospitals, Addis Ababa." Ethiopian Journal of Health Development **28**(1): 22-28.

Arrieta, A., A. García-Prado and J. Guillén (2011). "The Private Health Care Sector and the Provision of Prenatal Care Services in Latin America." World Development **39**(4): 579-587.

Bangladesh. National Institute of Population, R., Training, H. Bangladesh. Ministry of, W. Family, C. Associates for, R. Population and I. C. F. I. D. Program (2016). Bangladesh Health Facility Survey 2014. Final report, Dhaka, Bangladesh, NIPORT, 2015 Apr.**:** 276 p.

Danel, I. and G. Forgia (2005). "Contracting for basic health care in rural Guatemala - Comparison of the performance of three delivery models." Health Systems Innovations in Central America: Lessons and Impact of New Approaches: 49-88.

Farahbakhsh, M., H. Sadeghi-Bazargani, A. Nikniaz, J. S. Tabrizi, A. Zakeri and S. Azami (2012). "Iran's Experience of Health Cooperatives as a Public-Private Partnership Model in Primary Health Care: A Comparative Study in East Azerbaijan." Health Promot Perspect **2**(2): 287-298.

Khan, M. A., S. S. Owais, S. Ishaq, J. Walley, H. J. Khan, C. Blacklock, M. A. Khan and M. W. Azeem (2017). "Process evaluation of integrated early child development care at private clinics in poor urban Pakistan: a mixed methods study." BJGP Open **1**(3): bjgpopen17X101073.

Singh, K., I. Speizer, S. Handa, R. O. Boadu, S. Atinbire, P. M. Barker and N. A. Twum-Danso (2013). "Impact evaluation of a quality improvement intervention on maternal and child health outcomes in Northern Ghana: early assessment of a national scale-up project." Int J Qual Health Care **25**(5): 477-487.

Wallen, T., R. Blenden, I. Zafurallah, T. Vesel and R. Soto (2017). "Programmatic changes to reduce mortality and morbidity in humanitarian congenital heart surgery." Cardiology in the Young **27**(4): S227-S228.
